# Antitumor Activity of the Ethanolic Extract from *Syzygium aromaticum* in Colorectal Cancer Xenograft Mice

**DOI:** 10.3390/pharmaceutics18010079

**Published:** 2026-01-07

**Authors:** Thunyatorn Yimsoo, Weerakit Taychaworaditsakul, Hathaichanok Chuntakaruk, Worapapar Treesuppharat, Sumet Kongkiatpaiboon, Apipu Ariyachayut, Sunee Chansakaow, Teera Chewonarin, Parirat Khonsung, Seewaboon Sireeratawong

**Affiliations:** 1 Department of Pharmacology, Faculty of Medicine, Chiang Mai University, Chiang Mai 50200, Thailand; thunyatorn_y@cmu.ac.th (T.Y.); parirat.khons@cmu.ac.th (P.K.); 2 Laboratory Animal Center, Office of Advanced Science and Technology, Thammasat University, Pathum Thani 12120, Thailand; 3 Thammasat University Research Unit in Mechanisms of Drug Action and Molecular Imaging, Thammasat University, Pathum Thani 12120, Thailand; g4736963@gmail.com (W.T.); apipuluck@gmail.com (A.A.); 4 Department of Biochemistry, Faculty of Medicine, Chiang Mai University, Chiang Mai 50200, Thailand; weerakit.tay@cmu.ac.th (W.T.); teera.c@cmu.ac.th (T.C.); 5 Center for Artificial Intelligence in Medicine, Research Affairs, Faculty of Medicine, Chulalongkorn University, Bangkok 10330, Thailand; hathaichanok.c@chula.ac.th; 6 Center of Excellence in Computational Molecular Biology, Chulalongkorn University, Bangkok 10330, Thailand; 7 Drug Discovery and Development Center, Office of Advanced Science and Technology, Pathum Thani 12120, Thailand; s_u_m_e_t@hotmail.com; 8 Faculty of Pharmacy, Chiang Mai University, Chiang Mai 50200, Thailand; sunee.c@cmu.ac.th; 9 Clinical Research Center for Food and Herbal Product Trials and Development (CR-FAH), Faculty of Medicine, Chiang Mai University, Chiang Mai 50200, Thailand

**Keywords:** colorectal cancer, xenograft mice, *Syzygium aromaticum*, hydrodistillation residue of clove, metabolomic

## Abstract

**Background/Objectives**: Colorectal cancer (CRC) remains a leading cause of cancer-related mortality worldwide, and the development of effective therapies with improved safety profiles is urgently needed. The hydrodistillation residue extract of *Syzygium aromaticum* (SA) is rich in phenolic compounds, including ellagic acid and gallic acid, which are known for their antioxidant and anticancer properties. This study aimed to evaluate the anticancer efficacy, safety, and metabolic effects of SA extract in CRC models. **Methods**: The anticancer activity of SA was investigated using in vitro and in vivo approaches. Human colorectal cancer HCT116-Red-FLuc cells were used to assess cytotoxicity, selectivity, and dose- and time-dependent effects. In vivo efficacy was evaluated in a CRC xenograft mouse model using tumor volume measurement, micro-ultrasound imaging, and bioluminescence analysis. Hematological and blood biochemical parameters were analyzed to assess systemic safety. Untargeted metabolomic profiling was performed to explore metabolic alterations associated with SA treatment. **Results**: SA inhibited HCT116-Red-FLuc cell proliferation in a dose- and time-dependent manner and demonstrated selective cytotoxicity toward cancer cells, with a selectivity index of 4.41 at 24 h, although selectivity declined with prolonged exposure. In xenograft mice, SA significantly suppressed tumor growth and reduced metastatic incidence. The 500 mg/kg dose (SA500) showed the greatest antitumor efficacy while maintaining normal hematological and biochemical profiles, indicating a favorable safety margin compared with 5-fluorouracil (5FU). The 1000 mg/kg dose (SA1000) induced marked suppression of Ki-67, Bcl-2, and CD31 expression and enhanced apoptosis. Metabolomic analysis identified 44 differential metabolites related to fatty acid, amino acid, and nucleotide metabolism. **Conclusions**: These findings suggest that SA extract exerts significant antitumor activity against CRC with improved tolerability compared with conventional chemotherapy, supporting its potential as a complementary natural therapeutic candidate.

## 1. Introduction

Colorectal cancer (CRC) ranks among the most prevalent and lethal cancers worldwide. In Thailand, CRC represents the third most prevalent malignancy among males and the fourth among females, constituting a significant contributor to the national cancer burden [1]. The incidence of CRC among individuals younger than 50 years has risen markedly in recent years, suggesting that the disease is no longer limited to older age groups [2]. While conventional treatments such as surgery, chemotherapy, radiotherapy, and targeted therapies have contributed to lowering death rates in early-stage cases, their impact on advanced-stage disease remains limited. Patients treated with combinations of chemotherapy drugs like FOLFOX or FOLFIRI frequently suffer from side effects, including fatigue, gastrointestinal issues, and potential long-term organ complications [3,4,5,6]. These challenges, coupled with rising rates of resistance to chemotherapy, emphasize the urgent need for more effective and better-tolerated treatment alternatives. At present, there is an emerging concept of integrating herbal medicines with conventional pharmaceuticals to enhance therapeutic efficacy while minimizing the adverse effects associated with standard drug treatments [7]. Plant-derived compounds have remained a focal point in cancer research, with polyphenols drawing particular attention due to their antioxidant, anti-inflammatory, and antitumor properties [8,9,10].

*Syzygium aromaticum*, or clove, is known for its rich content of bioactive compounds, particularly gallic acid, ellagic acid, and eugenol [11,12]. Among these, gallic acid has attracted attention due to its ability to inhibit the migration of lung cancer cells and trigger apoptosis, reinforcing its potential as a natural agent in cancer prevention [13]. In one study, a nanoscale emulsion derived from clove bud essential oil displayed strong anticancer and antibacterial activity in vitro, primarily by inducing apoptosis and reducing cancer cell viability, suggesting its utility as a nanocarrier in cancer therapy [14]. Further analyses of clove flower bud extracts have confirmed the presence of gallic acid, ellagic acid, and eugenol, all of which showed cytotoxic effects against several cancer cell lines, including colon (HCT-116), breast (MCF-7), and liver (HepG2) cells. These results highlight the broad therapeutic potential of clove and its phenolic constituents [15].

Interestingly, although the hydrodistillation residue remaining after essential oil extraction from cloves is typically regarded as industrial waste, previous studies have demonstrated that this by-product still contains substantial levels of biologically active phenolic compounds, notably gallic acid and ellagic acid [16]. Importantly, the SA investigated in the present study is not a conventional clove extract, but an ethanolic extract obtained from this post-distillation residue through a secondary extraction process. Despite undergoing prior essential oil removal, the residue was found to retain detectable and reproducible phenolic markers with potential biological relevance. To date, the antitumor activity of such a post-distillation clove residue extract has not previously been evaluated in colorectal cancer animal models.

In more recent in vitro experiments, an ethanolic extract made from this residue (referred to as the SA extract) has demonstrated strong antioxidant activity and selective cytotoxicity toward colorectal cancer cells, especially the HCT116 cell line. Mechanistically, the SA extract was found to induce apoptosis via caspase-3/7 activation and promote sub-G1 cell cycle arrest by modulating key regulators such as p21 and cyclin D1 [16]. Together, these findings suggest that the extract could be a promising candidate for future preclinical evaluation.

In the present study, in vitro assays were conducted to evaluate the cytotoxic effects of the extract on HCT116-Red-FLuc colorectal cancer cells, as well as to determine their selectivity index by comparing the effects on cancer cells and normal lung fibroblasts. To further investigate its therapeutic potential, in vivo experiments were carried out using a xenograft mouse model, in which the extract was administered orally to assess its anti-tumor efficacy under physiological conditions. The xenograft model utilized HCT116-Red-FLuc cells, which express luciferase, enabling non-invasive, real-time monitoring of tumor progression through bioluminescence imaging [17]. Additionally, micro-ultrasound imaging was used to visualize tumor anatomy and measure volume, complementing both caliper-based measurements and bioluminescence data [18,19]. At the study endpoint, tumor tissues were analyzed via immunohistochemistry to evaluate the expression of key markers related to cell proliferation (Ki67), anti-apoptotic activity (Bcl-2), and angiogenesis (CD31). The TUNEL assay was also performed to confirm apoptotic cell death by detecting DNA fragmentation. To further explore treatment-associated effects, we conducted untargeted metabolomic profiling of plasma samples to identify systemic metabolic changes linked to the extract’s anticancer activity.

Metabolomics aims to comprehensively profile and quantify small endogenous metabolites within biological systems to reveal metabolic dysregulation associated with disease states [20,21]. As a key omics discipline, it complements genomics and proteomics by directly reflecting biochemical activities and physiological responses. Among numerous analytical platforms, liquid chromatography–tandem mass spectrometry (LC-MS/MS), particularly when coupled with a high-resolution quadrupole Orbitrap system, offers excellent sensitivity, specificity, and throughput, making it indispensable for metabolomics analysis [22]. The LC-MS/MS system can efficiently separate complex mixtures and detect a wide range of metabolites based on accurate mass and retention time, enabling comprehensive metabolic coverage with high reproducibility and resolution [23]. Therefore, LC-MS/MS-based metabolomics serves as a powerful tool to elucidate disease mechanisms, identify potential biomarkers, and support precision medicine research.

This integrative approach, which combines histological, protein expression, and metabolic analyses, provides a comprehensive assessment of the biological effects of SA extract and its potential as an alternative strategy for colorectal cancer (CRC) treatment. Furthermore, these findings may contribute to the future development of the extract as an adjunctive therapy to enhance the efficacy and safety of conventional chemotherapeutic regimens in CRC management.

## 2. Materials and Methods

### 2.1. Plant Materials, Extraction, and Phytochemical Analysis

The enriched phenolic extract of *S. aromaticum* (SA) used in this study was prepared according to our previous research [16]. *S. aromaticum* flower buds were purchased from local herb store in Chiang Mai, Thailand. The plant material was verified by a botanist at the Department of Pharmaceutical Sciences, Chiang Mai University, Thailand. A voucher specimen (SA012567) has been deposited at the Medicinal Plant Innovation Center, Faculty of Pharmacy, Chiang Mai University. *S. aromaticum*, commonly used in flower beds, is a spice with culinary and medicinal applications. *S. aromaticum* is used in cosmetics, medicine, gastronomy, and agriculture due to its rich variety of bioactive compounds, including gallic acid, flavonoids, eugenol acetate, and eugenol.

The extract was prepared from the hydrodistillation residue generated during clove essential oil production, which was collected and processed as a secondary raw material for extract development. Briefly, dried *S. aromaticum* samples were ground into a fine powder. It was first subjected to hydrodistillation. The marc from hydrodistillation was subjected to a successive extraction with ethyl acetate and ethanol using Soxhlet apparatus. Ethyl acetate extraction was performed using a Soxhlet apparatus to obtain the initial extract. After this process, the leftover residue from extraction with ethyl acetate was collected and further extracted with 95% ethanol. The extract was filtered through Whatman No.1 filter paper and concentrated using a rotary evaporator under reduced pressure. The ethanolic extract was labeled as the SA extract and was subsequently used in the experimental assays. Identification and quantification of major phytochemicals was done using LC-DAD-Q-Orbitrap-MS/MS. Prior to the experimental investigations in this study, the SA extract was reanalyzed to confirm the identification and quantification of its major phytochemical constituents using LC-DAD-Q-Orbitrap-MS/MS analysis.

A Vanquish UHPLC system (Thermo Fisher Scientific, Waltham, MA, USA) equipped with a Binary Pump F, Split Sampler FT, Column Compartment H, and a Diode Array Detector FG, all from Thermo Scientific (Waltham, MA, USA) was applied. The column was a Hypersil BDS C18 (Thermo Fisher Scientific, Waltham, MA, USA, 100 × 2.1 mm i.d., 2.4 µm). The mobile phases were (A) 0.1% formic acid in water and (B) methanol. A gradient program was set up at flow rate of 0.6 mL/min with 100% A for 3 min, then linear gradient elution from 0% to 40% B in A for 5 min, 40% B in A for 2 min, gradient from 40% to 90% B in A for 6 min, and 100% B for 4 min. Before each injection, the column was equilibrated with 100% A for 3 min. Column temperature was set at 25 °C with still air mode. Diode array detection was set at wavelengths of 254 and 272 nm. The injection volume was 2 µL for all samples and standards.

Mass spectrometry was analyzed in both positive and negative ionization modes, utilizing EASY-IC^TM^ internal mass calibration. The ion source was a Heated Electrospray Ionization (H-ESI). Spray voltage was set in the static mode at 3500 V for positive ions and 2500 V for negative ions. Nitrogen gas was applied in static mode with flow rates configured as follows: sheath gas at 60 arbitrary units (Arb), auxiliary gas at 15 Arb, and sweep gas at 2 Arb. The ion transfer tube temperature was 350 °C. The vaporizer temperature was set at 350 °C. Full-scan data were acquired over a mass range of 100–1000 *m*/*z* at a resolution of 60,000, with the RF lens set to 70%. Data-dependent MS/MS (ddMS^2^) acquisition was enabled with an intensity threshold of 5.0 × 10^5^. MS^2^ settings included an isolation window of 1.5 *m*/*z*, normalized collision energy, Orbitrap resolution of 15,000, and automatic scan range mode. Instrument operation and data acquisition were controlled using the Chromeleon™ Chromatography Data System software (version 7.3). Chromatograms were visualized and analyzed using FreeStyle software (version 1.8.65.0). Compound identification was performed by comparing acquired mass spectral data with reference spectra from the National Institute of Standards and Technology (NIST) and mzCloud databases, as well as previously published literature.

Quantitative analysis was based on the peak area obtained from a diode array detector. Standard gallic acid (**1**) and ellagic acid (**2**) were used. The calibration curves were constructed across a concentration range of 3.9–1000 µg/mL by plotting the peak area of gallic acid and ellagic acid against the corresponding concentrations. For sample preparation, SA extract was accurately weighed and extracted with a methanol–water mixture (1:1, *v*/*v*) to yield a concentration of 5 mg/mL.

### 2.2. In Vitro Assay

#### 2.2.1. Cell Culture

The HCT116-Red-FLuc cell line, a human colorectal cancer line possessing wild-type p53, was genetically modified to express the firefly luciferase gene from *Luciola italica* (Red-FLuc). This stably transfected luciferase-expressing cell line was obtained from PerkinElmer (Waltham, MA, USA). The normal human lung fibroblast line MRC-5 was procured from the American Type Culture Collection (ATCC, Manassas, VA, USA). HCT116-Red-FLuc cells were grown in McCoy’s 5A medium containing 10% fetal bovine serum (FBS), while MRC-5 cells were maintained in Dulbecco’s Modified Eagle Medium (DMEM) supplemented with 10% FBS and 1% penicillin–streptomycin. All cultures were kept at 37 °C in a humidified incubator with 5% CO_2_ atmosphere using 75 cm^2^ culture flasks.

#### 2.2.2. Cytotoxicity Assay

HCT116-Red-FLuc cells were plated in 96-well plates at a density of 5000 cells per well and allowed to attach for 24 h, while MRC-5 cells were seeded at 10,000 cells per well under the same incubation period. Following incubation, cells were exposed to either DMSO alone (vehicle control) or various concentrations of the SA extract dissolved in DMSO (3.13–800 µg/mL) for 24, 48, or 72 h. The final DMSO concentration in all wells did not exceed 0.8%. Cell viability was determined using the MTT assay. In brief, 20 µL of MTT solution (5 mg/mL) was added to each well and incubated for 3 h at 37 °C in a humidified 5% CO_2_ atmosphere. The supernatant was then removed, and 100 µL of DMSO was added to dissolve the formazan crystals. Absorbance was measured at 590 nm using a microplate spectrophotometer (Thermo Fisher Scientific, Vantaa, Finland), and cell viability was expressed as a percentage relative to the vehicle control. All experiments were performed in triplicates with three independent replicates. The half-maximal inhibitory concentration (IC_50_) values of SA against MRC-5 and HCT116-Red-FLuc cells were calculated using GraphPad Prism version 10.0.0 (GraphPad Software, Boston, MA, USA). The selectivity index (SI) was calculated to evaluate the preferential cytotoxicity of the extract toward cancer cells over normal cells. SI was determined as the ratio of IC_50_ in normal cells (MRC-5) to that in cancer cells (HCT116-Red-FLuc), according to the following formula:

SI = IC_50_ (MRC-5)/IC_50_(HCT116-Red-FLuc)

SI > 2 was considered an indicator of selective cytotoxicity of the SA extract toward cancer cells.

### 2.3. In Vivo Assay

#### 2.3.1. Animals, Housing, Diet, Water, and Ethic

All animal experiments were conducted in accordance with the ethical guidelines prescribed by the Animals for Scientific Purposes Act, B.E. 2558 (2015) of Thailand and the Guide for the Care and Use of Laboratory Animals, Eighth Edition, published by the National Research Council (NRC), Institute for Laboratory Animal Research (ILAR), USA. The study protocol was reviewed and approved by the Institutional Animal Care and Use Committee (IACUC) of Thammasat University, Pathumthani, Thailand (Approval No. 024/2024) and IACUC of Faculty of Medicine, Chiang Mai University, Chiang Mai, Thailand (Approval No. 10/2568). BALB/cAJcl-nu/nu male nude mice (6–8 weeks old) were obtained from Nomura Siam International Co., Ltd. (Bangkok, Thailand) and housed in individually ventilated cages (IVCs) under specific pathogen-free (SPF) conditions, which were maintained at 22 ± 1 °C and 30–70% relative humidity. Animals were acclimatized for 7 days prior to starting experiment and provided with autoclaved commercial rodent chow and sterile water ad libitum. Male mice were selected to minimize the influence of sex hormones on colorectal cancer development, as the incidence of this cancer is generally higher in males.

#### 2.3.2. Xenograft Mice Experiment

To evaluate the in vivo antitumor effect of the SA extract in a human colorectal cancer xenograft model, HCT116-Red-FLuc cells (5 × 10^5^ cells in 100 µL of serum-free McCoy’s 5A medium) were injected subcutaneously into the left flank of mice. Tumor growth was monitored, and once the tumors reached the desired volume, the mice were randomly assigned into four groups of six animals each. Group I (negative control, NC) received vehicle solution (10% DMSO in DPBS), Group II (positive control) was treated with 5-fluorouracil (5FU) at 20 mg/kg, Group III received the SA extract at 500 mg/kg (SA500), and Group IV received 1000 mg/kg (SA1000). All test compounds were freshly prepared in 10% DMSO in DPBS and administered orally once daily for 21 days.

#### 2.3.3. Tumor Volume, Body Weight and Organ Weight

The body weights and tumor volumes of the nude mice were regularly monitored throughout the experimental period. Tumor sizes were measured every three days by a digital caliper. Tumor volumes were calculated by the formula: ½ × length × width^2^ [24]. In addition, the mice underwent ultrasound and bioluminescence imaging once per week throughout the experimental period, as described below. Treatment was initiated once tumor volumes reached 50–100 mm^3^. Body weights were monitored weekly through the experiment. At the end of the study, all animals were humanely sacrificed using an overdose of isoflurane, followed by cervical dislocation to ensure death. The tumors, along with the lungs, liver, kidneys, and intestines, were then carefully removed and weighed.

#### 2.3.4. Micro-Ultrasound Imaging

The high-resolution ultrasound imaging was performed using the Vevo 3100 micro-ultrasound system (FUJIFILM VisualSonics Inc., Toronto, ON, Canada), equipped with MX250 (15–30 MHz) and MX550D (25–55 MHz) transducers for in vivo tumor assessment. Mice were anesthetized with isoflurane (2–3%) delivered through a nose cone and maintained under anesthesia throughout the imaging procedure. This non-invasive imaging modality enabled longitudinal monitoring of tumor progression and therapeutic response in the same animals, providing quantitative volume data without requiring euthanasia at each time point. Tumor imaging was conducted weekly during the experiment. Quantitative analysis of tumor volume was performed using Vevo LAB software version 5.6.0 (FUJIFILM VisualSonics Inc., Toronto, ON, Canada) by manually delineating tumor margins on serial image slices and calculating total volume through integrated 3D reconstruction algorithms.

#### 2.3.5. Bioluminescence Imaging

The bioluminescence imaging was carried out using IVIS Spectrum CT imaging system (Revvity, Waltham, MA, USA) to monitor tumor development in animals. For in vivo imaging, D-luciferin dissolved in DPBS was administered via intraperitoneal injection at a dose of 150 mg/kg, and mice were left for 20 min to allow the substrate distribution. Animals were anesthetized with 2–3% isoflurane and positioned inside the imaging chamber, where anesthesia was maintained throughout the imaging session. For ex vivo imaging, the same dose of D-luciferin solution was injected intraperitoneally into nude mice prior to euthanasia. Tumors and other organs were excised immediately and transferred into 12-well culture plates. Subsequently, D-luciferin solution (300 μg/mL in DPBS) was added to fully cover the organs before imaging. Bioluminescent signals were captured as color images and overlaid on white light images. Signal intensity was quantified in a unit of photons/sec using Living Image software version 4.7.4 (Revvity, Waltham, MA, USA). A signal at least 3- to 5-fold above background is typically considered as true positive, particularly in tumor xenograft models [25]. Bioluminescent intensity was analyzed as average surface radiance (photons/sec/cm^2^/sr) to detect cancer cells, characteristics of tumor growth, and metastasis for more accurate determination of internal bioluminescent sources [26].

#### 2.3.6. Hematological, Blood Chemical and Histopathology Analysis

Blood samples were collected for hematological and blood chemical analyses. Complete blood count (CBC) parameters were determined using an automated hematology analyzer (ABX Micros ESV60, HORIBA, Irvine, CA, USA). Serum biochemical parameters, including aspartate aminotransferase (AST), alanine aminotransferase (ALT), blood urea nitrogen (BUN), and creatinine, were measured using a DRI-CHEM NX500 automated analyzer (FUJIFILM, Tokyo, Japan). For histopathological examination, liver tissues were fixed, embedded in paraffin, and sectioned at a thickness of 5 μm. The sections were stained with hematoxylin and eosin (Leica Biosystems, Nussloch, Germany), followed by dehydration and mounting. Histopathological alterations were subsequently examined under a light microscope bright-field microscope (Olympus BX53, Olympus Corporation, Tokyo, Japan) to assess tissue morphology and lesion characteristics.

### 2.4. Immunohistochemistry and TUNEL Assay

#### 2.4.1. Immunohistochemistry of Ki67, Bcl-2, and CD31

The proliferative activity, anti-apoptotic signaling, and vascularization of tumor tissues were examined by immunohistochemistry. Formalin-fixed, 5 μm-thick sections of paraffin-embedded tumor tissue were subjected to antigen retrieval using an EDTA-based buffer (pH 8.0) for 25 min in a Leica Bond automated stainer (Leica Biosystems, Nussloch, Germany), followed by quenching of endogenous peroxidase activity for 5 min. Sections were incubated with the following primary antibodies for 40 min: a monoclonal mouse anti-Ki-67 (clone MIB-1, 1:300, DAKO A/S, Glostrup, Denmark), a monoclonal mouse anti-Bcl-2 (clone 124, 1:1500, Cell Marque, Rocklin, CA, USA), and a monoclonal mouse anti-CD31 (clone JC/70A, 1:800, DAKO/Agilent Technologies, Santa Clara, CA, USA). Positive controls included appendix tissue for Ki-67 and tonsil tissue for Bcl-2 and CD31. After incubation with a post-primary reagent (10 min) and polymer (10 min), chromogenic detection was performed using 3,3′-diaminobenzidine (DAB) (Leica Biosystems, Nussloch, Germany) for 3 min, and counterstaining was carried out with Hematoxylin 560 (Leica Biosystems, Nussloch, Germany) for 15 min. Slides were dehydrated, cover slipped, and examined in bright-field mode using a Lionheart FX Automated Microscope (BioTek Instruments, Agilent Technologies, Santa Clara, CA, USA). Ki-67 and Bcl-2 expression levels were quantified by calculating the percentage of positively stained nuclei relative to the total number of tumor cells. For CD31 staining, microvessel density was assessed by counting the number of distinct CD31-positive vessels, and the mean value was recorded for each tumor. Images were acquired at ×10 and ×20 magnifications.

#### 2.4.2. TUNEL Assay for Detection of Apoptotic DNA Fragmentation

Tumor tissues were further processed for TUNEL assays. Apoptotic cells in paraffin-embedded tumor tissues were detected using the TUNEL Assay Kit, HRP-DAB (Abcam; ab206386, Cambridge, UK) according to the manufacturer’s instructions. Briefly, 10 μm-thick sections of paraffin-embedded tumor tissue from mice sections were deparaffinized in xylene, rehydrated through graded ethanol, and permeabilized with Proteinase K (1:100 in dH_2_O) for 20 min at room temperature. Endogenous peroxidase activity was quenched with 3% hydrogen peroxide in methanol for 5 min. After equilibration in TdT Equilibration Buffer (30 min), slides were incubated with TdT Labeling Reaction Mix for 90 min at 37 °C in a humidified chamber. The reaction was terminated with Stop Buffer, and sections were blocked with Blocking Buffer for 10 min. Biotin-labeled DNA was detected using HRP-conjugated streptavidin (1:25 in Blocking Buffer, 30 min), followed by visualization with DAB chromogen (15 min), producing brown precipitates at sites of DNA fragmentation. Counterstaining was performed with methyl green (2 min), and slides were dehydrated through ethanol, cleared in xylene, and mounted with coverslips [27]. Positive controls were treated with DNase I to induce DNA strand breaks, and negative controls were prepared by omitting the TdT enzyme. Stained sections were examined under a bright-field microscope (Olympus BX53, Olympus Corporation, Tokyo, Japan) equipped with a DP74 digital camera. Images were acquired at ×10 magnifications. The proportion of TUNEL-positive nuclei was quantified using ImageJ software (version 1.54g; NIH, Bethesda, MD, USA) and expressed as a percentage of total nuclei.

### 2.5. Metabolomic Profile Analysis

Mice’s plasma was used for metabolomic profiling. At the end of the experimental period, whole blood was collected from mice via cardiac puncture under terminal anesthesia and transferred into heparin-coated tubes. The samples were centrifuged at 4000× *g* for 5 min at room temperature to separate plasma, which was then aliquoted and stored at −80 °C until analysis. For metabolomic analysis, plasma samples were prepared using a protein precipitation method. Briefly, 50 µL of plasma was mixed with 150 µL of a mixture of acetonitrile-methanol-formic acid (75:25:0.2, *v*/*v*/*v*), vortexed for 1 min, sonicated for 10 min and froze at −20 °C for 30 min to precipitate proteins. The mixture was then centrifuged at 13,000 rpm for 10 min at 4 °C, and the supernatant was collected and filtered through 0.22 µm-nylon membrane filter. Untargeted metabolomic profiling was performed using liquid chromatography coupled with tandem mass spectrometry (LC-MS/MS). The LC-MS/MS analysis was done on a Vanquish UHPLC system (Thermo Fisher Scientific, Waltham, MA, USA) equipped with a Binary Pump F, Split Sampler FT, Column Compartment H, and a Diode Array Detector FG. The column was a HILIC Waters XBridge Amide column (100 × 4.6 mm i.d., 3.5 µm). The mobile phases were (A) 20 mM ammonium acetate in water and (B) acetonitrile. A mobile-phase time program was set up at a constant flow rate of 1 mL/min with 95% B in A for 6 min, then linear gradient elution from 95% to 85% B in A for 6 min, 85% to 40% B in A for 3 min, 40% to 5% B in A for 1 min, and 5% B in A for 2 min. The column was equilibrated with 95% B in A for 5 min before injection. Column temperature was set at 25 °C with still air mode. The injection volume was 2 µL for all samples and standards. Mass spectrometric analysis was conducted in both positive and negative ionization modes, utilizing EASY-IC^TM^ internal mass calibration. The ion source was a Heated Electrospray Ionization (H-ESI). Spray voltage was set in the static mode at 3500 V for positive ions and 2500 V for negative ions. Nitrogen gas was applied in static mode with flow rates configured as follows: sheath gas at 60 arbitrary units (Arb), auxiliary gas at 15 Arb, and sweep gas at 2 Arb. The ion transfer tube and vaporizer temperatures were both maintained at 350 °C. Full-scan data were acquired over a mass range of 100–1000 *m*/*z* at a resolution of 60,000, with the RF lens set to 70%. Data-dependent MS/MS (ddMS^2^) acquisition was enabled with an intensity threshold of 5.0 × 105. MS^2^ settings included an isolation window of 1.5 *m*/*z*, normalized collision energy, Orbitrap resolution of 15000, and automatic scan range mode. Instrument operation and data acquisition were controlled using the Chromeleon™ Chromatography Data System software. Chromatograms were visualized and analyzed using FreeStyle software. Compound identification was performed by Compound Discoverer 3.3.3.200.

### 2.6. Statistical Analysis

Statistical analyses were conducted using SPSS Statistics version 25 (SPSS Inc., Chicago, IL, USA). Data are presented as mean ± standard error of the mean (S.E.M). The Shapiro–Wilk test was applied to assess data normality. For datasets following a normal distribution, one-way analysis of variance (ANOVA) followed by Tukey’s post hoc multiple comparison test was performed. In contrast, non-normally distributed data were analyzed using the Kruskal–Wallis nonparametric ANOVA followed by Dunn’s post hoc test. Statistical significance was defined as *p* < 0.05. For multivariate evaluations, the normalized peak intensities of metabolites from all analytical platforms were analyzed using the multivariate software MetaboAnalyst 6.0 [28]. The software was then used for cluster analyses, including principal component analysis (PCA) and heatmap analyses, with Pareto scaling applied.

## 3. Results

### 3.1. SA Extract Preparation and Characterization

**Figure 1:**
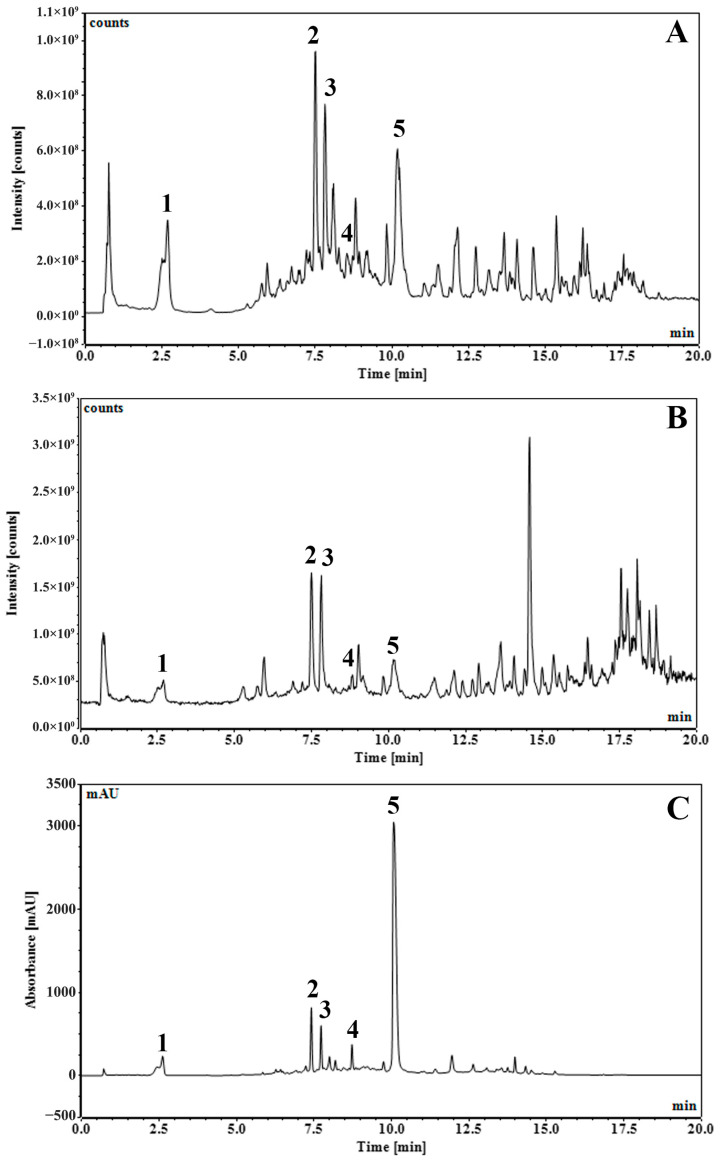
UHPLC-DAD-MS/MS chromatograms of *S. aromaticum* flower bud extract of residue from hydrodistillation (SA) with total ion counts of negative mode with full scan at 100–1000 *m*/*z* (**A**), total ion counts of positive mode with full scan at 100–1000 *m*/*z* (**B**), and UV 254 nm (**C**).

**Figure 2:**
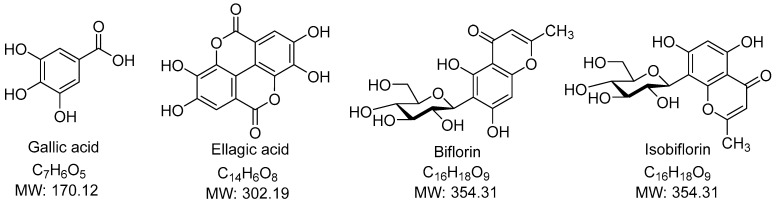
Chemical Structure of identifiable compounds.

The residue of *S. aromaticum* flower buds left over after hydrodistillation was processed to obtain an enriched phenolic extract (SA extract). It is a common waste after producing clove bud oil, although useful phenolic constituents remain. The Soxhlet extraction using ethyl acetate of the residue of *S. aromaticum* flower buds left over after hydrodistillation can remove the remaining eugenol and other terpenoid residues. Then, extraction of ethyl acetate marc using ethanol can yield phenolics as major constituents. The major components of SA extract were identified and quantified by LC-DAD–MS/MS methods. The predominant constituents identified in SA extract were gallic acid and ellagic acid. Representative chromatograms are shown in Figure 1. Peak identification is demonstrated in Table 1 and the chemical structures of identifiable compounds are illustrated in Figure 2. A list of mass spectra for each peak is shown in Supplementary Figures S1–S5.

The predominant constituents in the SA extract were gallic acid (**1**), bioflorin and isobioflorin (**2**, **3**) and ellagic acid (**5**), accompanied by some unidentified compounds. Gallic acid (**1**) and ellagic acid (**5**) could be explicitly identified from their parent ions. Gallic acid (**1**) had [M−H]^−^ ion at *m*/*z* of 169.0141 Da (calculated 169.10315 Da) in negative mode and [M+H]^+^ of 171.0285 Da (calculated 171.02880 Da) in positive mode. Neutral loss of CO_2_ (44 Da) due to the α mechanism in gallic acid (**1**) was confirmed by the fragment ions at *m*/*z* 125 in negative mode and *m*/*z* 127 in positive mode. Ellagic acid (**5**) had [M−H]^−^ ion at *m*/*z* 300.9986 Da (calculated 300.99789 Da), [M+H]^+^ ion at *m*/*z* 303.01354 Da (calculated 303.01354 Da), and [M+Na]^+^ ion at *m*/*z* 324.9954 (calculated 324.99549 Da). Neutral loss of CO_2_ (44 Da) due to the α mechanism in ellagic acid (**5**) was confirmed by the fragment ions at *m*/*z* 257 in negative mode. Identification of gallic acid and ellagic acid was also confirmed using co-chromatography with their authentic standards. Biflorin and isobiflorin (**2**, **3**) could tentatively be identified from [M−H]^−^ ions at an *m*/*z* of 353.0869 or 353.0870 (calculated 353.08671) and [M+H]^+^ ions at an *m*/*z* of 355.1018 or 355.1019 (calculated 355.10236). The complete identification of bioflorin isomers could not be done, which required authentic compounds and is a limitation of our study. The results were well in line with our previous study [16].

Standardization was performed against selected marker compounds (e.g., gallic acid and ellagic acid) quantified using peak area from the LC–DAD method. External standard calibration was done using authentic compounds and described in our previous work [16]. Quantitative analysis showed that the SA extract contained gallic acid and ellagic acid at concentrations of 2.64% and 11.87% *w*/*w*, respectively. For the current study, the specific batch of extract used was comparable with the previously characterized reference batch.

### 3.2. In Vitro Assay

#### 3.2.1. Effects of SA Extract on Cell Viability in HCT116-Red-FLuc Cells

**Figure 3:**
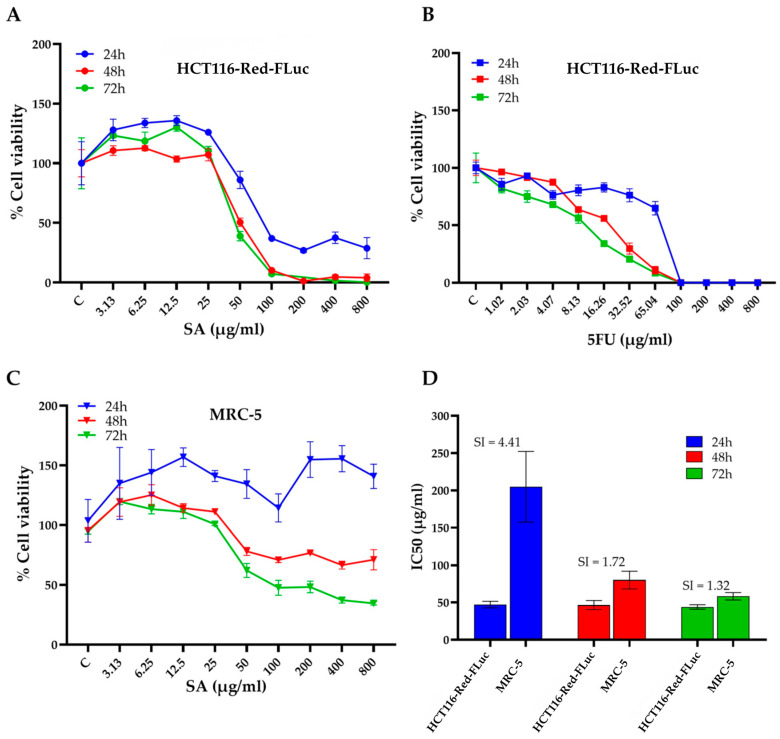
Cytotoxic effects and selectivity of SA extract against HCT116-Red-FLuc colorectal cancer cells and normal MRC-5 fibroblasts. (**A**) Cell viability of HCT116-Red-FLuc cells treated with increasing concentrations of SA extract for 24, 48, and 72 h, assessed by MTT assay. A dose- and time-dependent decrease in cell viability was observed, with greater cytotoxicity at longer incubation times. (**B**) Cytotoxic effects of 5FU in HCT116-Red-FLuc cells, shown as dose–response curves at 24, 48, and 72 h. The concentration range of 5-FU was selected based on preliminary in vitro cytotoxicity screening, as higher concentrations resulted in near-complete cell death. (**C**) Cell viability of normal MRC-5 fibroblasts exposed to SA extract for 24, 48, and 72 h. Compared to HCT116-Red-FLuc, MRC-5 cells were less sensitive to SA-induced cytotoxicity. (**D**) IC_50_ values of SA extract in HCT116-Red-FLuc and MRC-5 cells at each time point, with calculated selectivity index (SI = IC_50_ MRC-5/IC_50_ HCT116). The highest selectivity was observed at 24 h (SI = 4.41), indicating a preferential cytotoxic effect toward cancer cells. Data are presented as the mean ± standard deviation (SD) obtained from three independent experiments.

The cytotoxic potential of *S. aromaticum* hydrodistillation residue extract (SA) was assessed in HCT116-Red-FLuc colorectal cancer cells using the MTT assay at 24, 48, and 72 h (Figure 3A). SA treatment resulted in a clear dose- and time-dependent reduction in cell viability. At 72 h, marked cytotoxic effects were observed at concentrations ≥ 25 µg/mL, with near-complete inhibition achieved at concentrations of 200 µg/mL and above, beyond which no substantial further reduction in cell viability was observed. Similarly, at 48 h, 200 µg/mL was sufficient to achieve maximal cytotoxic effect, with higher concentrations showing comparable levels of inhibition. In contrast, minimal effects on cell viability were detected after 24 h of exposure, suggesting that prolonged treatment enhances the cytotoxic activity of the extract. For comparison, the standard chemotherapeutic agent 5-fluorouracil (5FU) was tested under similar conditions (Figure 3B). 5FU also exhibited a dose- and time-dependent inhibition of cell viability in HCT116-Red-FLuc cells, with a more rapid onset of action compared to SA. Marked cytotoxicity was observed as early as 24 h post-treatment, and cell viability was nearly eliminated at concentrations ≥ 25 µg/mL by 72 h. These findings indicate that while 5FU exerts potent and rapid cytotoxicity, SA extract demonstrates comparable efficacy at later time points, suggesting potential as a time-dependent anticancer agent.

#### 3.2.2. IC_50_ Determination and Selectivity Index (SI) of SA Extract

To further evaluate the cytotoxic specificity of SA extract, the half-maximal inhibitory concentration (IC_50_) values were determined in both HCT116-Red-FLuc cells and non-cancerous MRC-5 human lung fibroblasts after 24, 48, and 72 h of treatment (Table 2) In HCT116-Red-FLuc cells, the IC_50_ values were 47.16 ± 4.39 µg/mL (24 h), 46.61 ± 6.01 µg/mL (48 h), and 44.12 ± 2.99 µg/mL (72 h). In MRC-5 cells had IC_50_ values that were markedly higher at 204.78 ± 47.39 µg/mL, 80.09 ± 11.82 µg/mL, and 58.33 ± 4.95 µg/mL, respectively. The selectivity index (SI), calculated as the ratio of IC_50_ in MRC-5 to HCT116 cells, was used to evaluate preferential cytotoxicity towards cancer cells. The highest SI was observed at 24 h (SI = 4.41), indicating a favorable therapeutic window. However, the SI decreased to 1.72 and 1.32 at 48 and 72 h, respectively, reflecting a time-dependent decline in selectivity (Figure 3D).

### 3.3. In Vivo Assay

**Figure 4:**
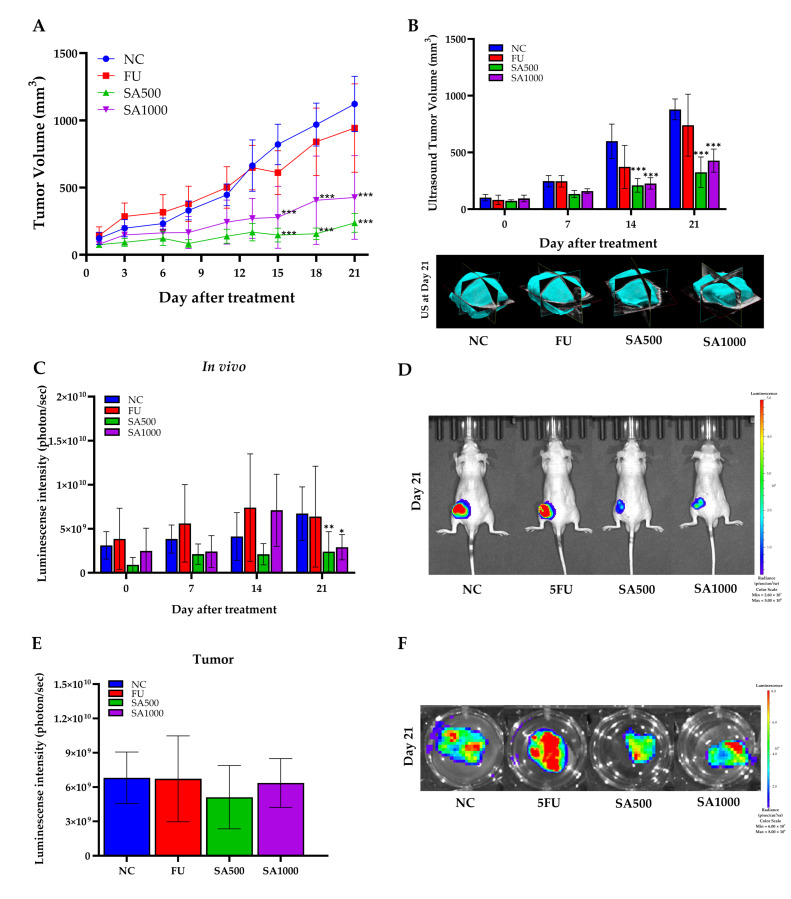
Antitumor effects of SA extract compared with 5-fluorouracil (5FU) in HCT116-Red-FLuc xenograft mice. (**A**) Tumor growth curve measured by caliper every 3 days post-treatment with SA at 500 and 1000 mg/kg, 5FU, or negative control (NC). Tumor volume was significantly suppressed in SA-treated groups compared to NC. (**B**) Ultrasound-based tumor volume assessment on days 0, 7, 14, and 21, with representative 3D reconstructions at day 21. SA-treated groups showed significant tumor volume reduction compared to NC. (**C**) Quantitative in vivo bioluminescence imaging showing tumor-derived luminescence intensity (photons/sec) at different time points. A marked reduction in luminescence signal was observed in SA500 on day 21. (**D**) Representative bioluminescence images of live mice on day 21 indicate reduced tumor burden in SA- and 5FU-treated groups compared to NC. (**E**) Ex vivo bioluminescence quantification from excised tumors at day 21 confirmed reduced tumor viability in both SA- and 5FU-treated mice. (**F**) Representative ex vivo bioluminescence images of excised tumors at day 21 corroborated the in vivo observations. Data are expressed as mean ± SD (*n* = 5 per group). Statistical significance versus NC: * *p* < 0.05, ** *p* < 0.01, *** *p* < 0.001.

Tumor progression was monitored using three complementary approaches: digital caliper measurements, high-resolution micro-ultrasound imaging, and bioluminescence imaging (Figure 4). As shown in Figure 4A, caliper-based tumor volume analysis indicated that 5FU treatment partially inhibited tumor growth compared to NC, particularly evident from day 12 onwards. However, both SA-treated groups demonstrated superior tumor suppression compared to 5FU. Importantly, SA500 exhibited the most pronounced effect, significantly reducing tumor volume from day 14 onward (*p* < 0.001), while SA1000 also suppressed tumor growth but to a slightly lesser extent than SA500. Tumor volume in the 5FU group remained consistently higher than in both SA-treated groups, suggesting limited efficacy under the current dosing regimen. Consistent with these findings, Figure 4B shows that ultrasound-derived tumor volume measurements revealed a similar trend. On day 21, mice in the 5FU group displayed moderate tumor reduction compared to NC. However, tumor volumes remained significantly greater than those in SA500 and SA1000 groups (*p* < 0.001). Representative 3D ultrasound images at day 21 further confirmed the limited tumor regression in 5FU-treated mice compared to the marked shrinkage observed in SA-treated groups. To further assess tumor viability, bioluminescence imaging was performed at serial time points (Figure 4C). While the 5FU group showed reduced luminescence intensity at day 21 compared to NC, the reduction did not reach statistical significance. In contrast, the SA500 group exhibited a statistically significant decrease in photon flux (*p* < 0.01), reflecting reduced tumor cell viability. The SA1000 group also showed a declining trend. Representative bioluminescence images (Figure 4D) on day 21 visually support these quantitative results, with stronger luminescent signals observed in the NC and 5FU groups compared to the weaker or absent signals in SA-treated groups. To validate these findings, ex vivo bioluminescence imaging was performed on excised tumors on day 21. Quantitative analysis (Figure 4E) revealed markedly lower photon emission from tumors in both SA500 and SA1000 groups relative to NC and 5FU, with the SA500 group showing the lowest average signal intensity. This pattern was consistent with the representative ex vivo images (Figure 4F), in which tumors from SA-treated mice exhibited notably reduced bioluminescent signal, indicating diminished tumor cell activity. Collectively, these data confirm that SA extract, particularly at 500 mg/kg, exerts potent antitumor effects by inhibiting tumor growth and reducing tumor cell viability more effectively than 5FU in this xenograft model.

**Figure 5:**
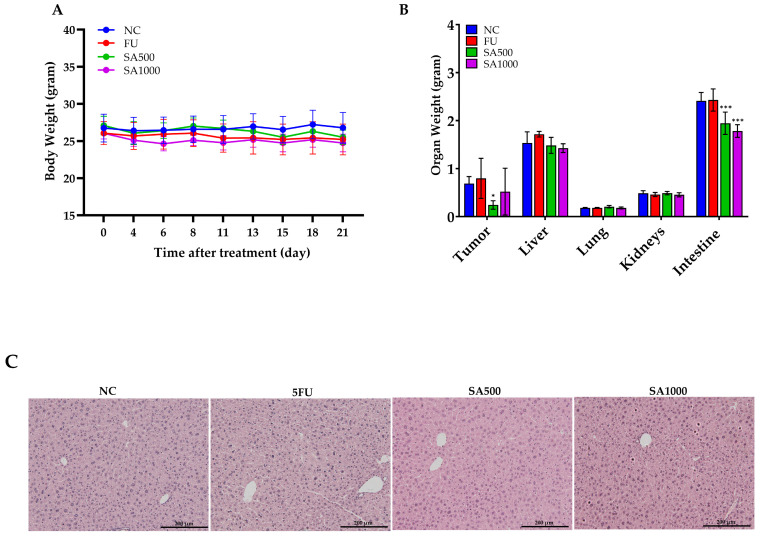
Evaluation of body weight, organ weight, and hepatic histology following treatment with 5FU and SA extract in HCT116-Red-FLuc xenograft-bearing nude mice. (**A**) Body weight was monitored throughout the 21-day treatment period. No significant changes were observed among the treatment groups, indicating the absence of overt systemic toxicity. (**B**) Organ weights at necropsy on day 21. Tumor weight was significantly reduced in all treatment groups compared to the negative control (NC), with the lowest weight observed in the SA500 group. Intestine weight was significantly decreased in the SA500 and SA1000 groups, while weights of the liver, lungs, and kidneys remained unchanged across groups. (**C**) Histological examination of liver tissue demonstrated normal morphology in all groups (hematoxylin and eosin staining, 10×). Data are presented as mean ± SD (*n* = 5 per group). Statistical significance versus NC: * *p* < 0.05, *** *p* < 0.001.

To evaluate the safety effects associated with SA extract and 5FU treatment, body weight, organ weights, and blood parameters were monitored throughout and at the end of the experimental period. As shown in Figure 5A, body weight remained stable in all groups during the 21-day treatment, indicating no overt systemic toxicity from SA extract or 5FU. Organ weight assessments at necropsy day (Figure 5B) showed a significant reduction in tumor mass in all treatment groups compared to NC, with SA500 producing the lowest tumor weight. No differences were observed in liver, lung, or kidney weights, while intestine weight was significantly decreased in the SA500 group (*p* < 0.001). Moreover, ex vivo bioluminescence analysis further substantiated the effects of the treatments on metastatic dissemination. Notably, brain metastases were observed exclusively in the 5FU-treated mice, whereas no such lesions were detected in either of the SA groups (Supplementary Figures S6 and S7). For hematological and blood chemical parameters, the 5FU-treated group showed slight reductions in red blood cell (RBC), hemoglobin (HGB), hematocrit (HCT), and platelet (PLT) counts, as shown in Table 3. In contrast, hematological values in the SA500-treated group were comparable to those of the normal control (NC), whereas the SA1000-treated group exhibited a slight decrease in platelet count. Regarding liver function, alanine aminotransferase (ALT) levels were lowest in the SA500 group. No significant alterations in renal function parameters were observed across all treatment groups. Nevertheless, all hematological and blood chemical values that showed variations remained within normal range. The findings revealed normal hepatic morphology and well-preserved tissue morphology across all experimental groups (Figure 5C).

### 3.4. Immunohistochemistry and TUNEL Assay

**Figure 6:**
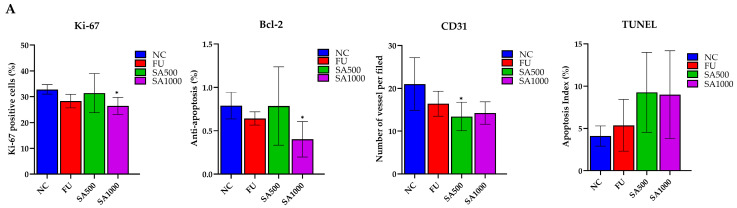

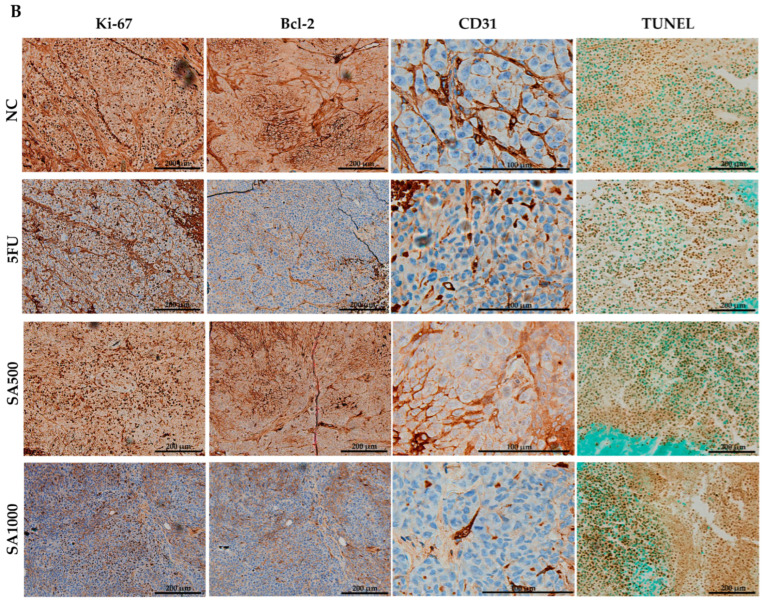
Immunohistochemical and TUNEL analysis of colorectal cancer xenograft tumors. (**A**) Quantification of Ki-67–positive cells, Bcl-2 expression, CD31-positive microvessels, and TUNEL-positive nuclei in tumors from the NC, 5FU, SA1000, and SA500 groups. Data are presented as mean ± SD. (**B**) Representative IHC and TUNEL staining images. Tumors from the NC group exhibited strong Ki-67, Bcl-2, and CD31 staining with few TUNEL-positive cells. In contrast, treatment with 5-FU and SA1000 markedly reduced Ki-67, Bcl-2, and CD31 expression while increasing TUNEL positivity, whereas SA500 produced moderate effects relative to NC. Scale bars: 200 μm (Ki-67, Bcl-2, and TUNEL) and 100 μm (CD31). Statistical significance versus NC: * *p* < 0.05.

Cell proliferation in excised tumors was evaluated by Ki-67 immunostaining. Tumors in the NC group displayed strong nuclear Ki-67 staining, indicating high proliferative activity. By contrast, 5FU and SA500 treatment markedly reduced the proportion of Ki-67–positive cells. However, a significant reduction was observed in the SA1000 group, with staining levels comparable to those in the NC group. In addition, assessment of the anti-apoptotic marker Bcl-2 revealed strong immunoreactivity in tumor cells of the NC group. Following treatment, 5FU markedly diminished Bcl-2 expression. A slight decrease was observed in the SA500-treated group compared with the NC group, whereas the SA1000-treated group showed a statistically significant and more pronounced reduction in Bcl-2 expression. These findings indicate that SA1000 more effectively suppressed anti-apoptotic signaling compared with the other groups. Angiogenesis was evaluated by CD31 immunostaining to assess tumor vascularization. Tumors in the NC group displayed a high density of microvessels, while treatment with 5FU resulted in a marked reduction in vascular structures. The SA500-treated group showed a statistically significant decrease in microvessel formation compared with the NC group, whereas the SA1000-treated group exhibited a pronounced but statistically non-significant decline in CD31-positive vessels. Collectively, these results indicate that SA500 possesses a more potent anti-angiogenic activity than the other treatments, highlighting its superior ability to suppress neovascularization within the tumor microenvironment. Finally, the TUNEL assay further verified apoptosis by detecting DNA fragmentation, a characteristic feature of programmed cell death. Only a few TUNEL-positive nuclei were detected in the NC group, indicating low basal apoptotic activity. In contrast, 5FU treatment markedly increased the number of apoptotic cells. Both SA500 and SA1000 groups exhibited a pronounced induction of apoptosis, which was markedly greater than that observed in the NC and 5FU groups (Figure 6).

### 3.5. PCA Reveals Distinct Metabolite Profiles Across Treated and Control Groups

**Figure 7:**
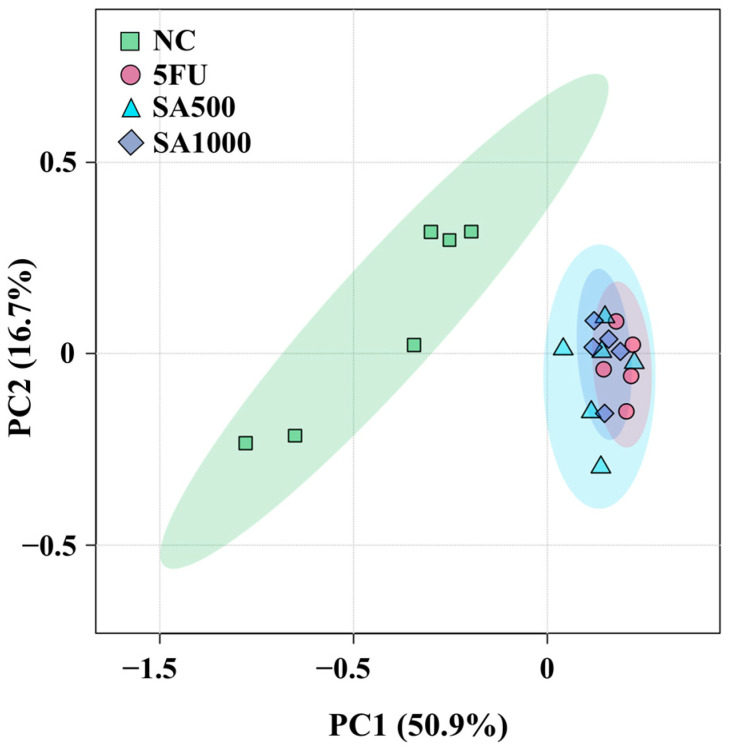
Principal component analysis (PCA) score plots of all groups (NC, 5FU, SA500, and SA1000) obtained using liquid chromatography coupled with tandem mass spectrometry (LC-MS/MS) on an Orbitrap mass spectrometer. Each point represents a biological replicate (*n* = 6, 5, 6, and 5, respectively).

To provide an overview of the differences in metabolite profiles among the four treatment groups, an unsupervised principal component analysis (PCA) was per-formed using metabolite peak areas obtained from the analytical platform (Figure 7). Principal components 1 and 2 accounted for 50.9% and 16.7% of the total variance, respectively, based on data acquired via liquid chromatography coupled with tandem mass spectrometry (LC-MS/MS) on an Orbitrap mass spectrometer. The 5FU, SA500, and SA1000 groups were positioned on the positive side of PC1, whereas the NC group was located on the negative side, indicating that the metabolite profiles of the treated groups were more similar to one another than to those of the negative control (NC) group. Variables contributing to PC2 did not clearly separate the treatment groups, as the 5FU, SA500, and SA1000 clusters partially overlapped, suggesting comparable metabolic responses among these treatments. Notably, PCA revealed considerable metabolic variation across all groups, with the treated groups showing partial overlap. The NC group also showed variability among replicates, with some samples clustering more closely than others. Overall, the differences in metabolite profiles among the treatment groups were captured by PCA. Heatmap analyses were conducted to further identify the specific metabolites contributing to the observed variation.

### 3.6. Heatmap Analysis Illustrates Changes in Metabolite Concentrations

**Figure 8:**
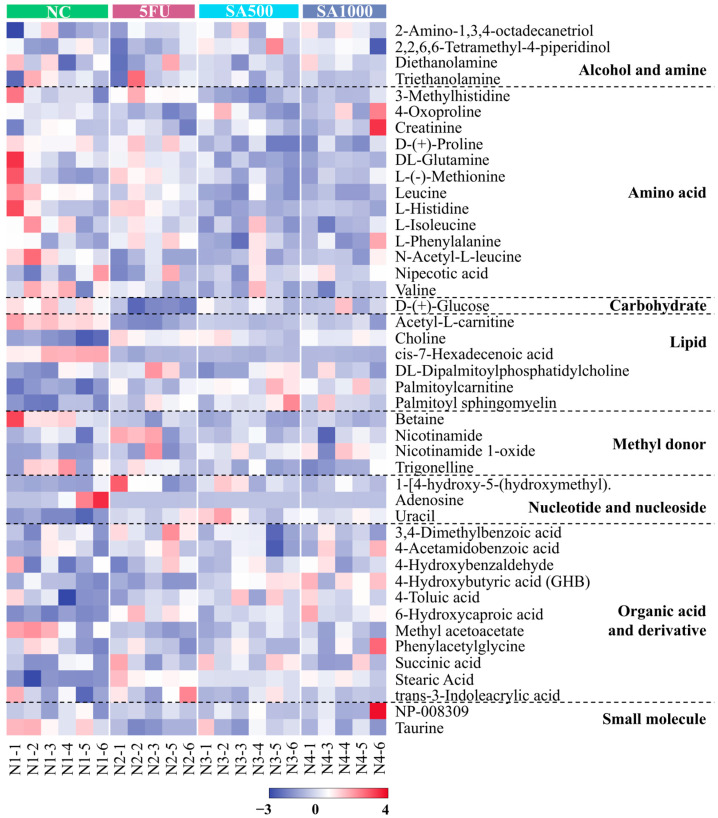
Heatmap visualization of the top 44 annotated metabolites is shown based on their relative abundance. The metabolites were measured using liquid chromatography coupled with tandem mass spectrometry (LC-MS/MS) on an Orbitrap mass spectrometer. Data are shown across tumor-bearing nude mice groups under different treatments (NC, 5FU, SA500, and SA1000). The color scale indicates the relative fold change of each metabolite between groups, with red and purple representing higher and lower abundances, respectively.

The top 44 metabolites annotated from the analytical platform using liquid chromatography coupled with tandem mass spectrometry (LC-MS/MS) on an Orbitrap mass spectrometer were selected based on their relative abundance for visualization in all tumor-bearing nude mice under different treatments (NC, 5FU, SA500, and SA1000) shown as Figure 8. The heatmap was organized into classes according to the involvement of these metabolites in various metabolic pathways.

## 4. Discussion

The hydrodistillation residue extract of *S. aromaticum* (SA) used in this study was comprehensively characterized using LC–MS/MS, a gold-standard technique for the identification and quantification of phenolic compounds [30]. The tested extract was derived from the same production batch as previously reported in our publication [16], which confirmed the presence of gallic acid and ellagic acid as major bioactive constituents. The LC–MS/MS analysis identified these major compounds, known phenolics with established anticancer activity in several tumor types, including colorectal cancer (CRC). In this study, ellagic and gallic acid were identified as quality-control markers of the extract. Both compounds are well-documented phenolics with reported anticancer activities. Ellagic acid has been shown to inhibit multiple oncogenic pathways and exert antiproliferative and pro-apoptotic effects in cervical, gastric, hepatocellular, and colorectal cancer models [31]. Similarly, gallic acid inhibits colorectal cancer cell proliferation, migration, and invasion and suppresses tumor growth and liver metastasis in xenograft models [32]. Moreover, other studies suggest that crude botanical extracts may exhibit greater anticancer activity than individual compounds, potentially due to additive or synergistic interactions among multiple bioactive constituents [33,34]. Therefore, further in vitro and in vivo studies using the standardized extract as well as ellagic acid and gallic acid should be carried out to clarify their relative efficacy, safety, and underlying mechanisms of action.

Interestingly, SA showed slower, progressive cytotoxicity compared to 5FU, consistent with the multitarget action of its phenolic compounds. Ellagic acid, for instance, gradually modulates oxidative stress and apoptosis in cell and animal models [35], while gallic acid are known to regulate oxidative stress, proliferation, and apoptosis gradually rather than causing immediate cytotoxicity [36,37]. These findings suggest that delayed SA activity results from pathway modulation by its active compounds rather than direct cytotoxicity, a typical feature of natural substances acting on multiple cellular pathways [38,39]. This agrees with our previous finding that SA extract modulates multiple pathways by suppressing proliferation and inducing apoptosis in colorectal cancer cells [16]. Together, these findings suggest that the SA extract acts through a gradual, multi-pathway regulatory mechanism characteristic of natural phenolic mixtures, leading to sustained antiproliferative and pro-apoptotic effects in CRC.

The IC_50_ data indicate that the SA extract exhibits greater cytotoxicity toward cancer cells compared to normal fibroblasts, particularly during the initial phases of treatment. After 24 h, the selectivity index (SI) was calculated at 4.41, suggesting a promising therapeutic margin. An SI exceeding two is commonly taken as an acceptable threshold for selective anticancer activity [40,41]. However, this selectivity decreased over time, with SI values dropping to 1.72 at 48 h and 1.32 by 72 h. This observed trend may reflect the accumulation of cellular stress or unintended cytotoxic effects on normal cells with prolonged exposure. These findings underscore the importance of optimizing treatment duration and dosage to maximize anticancer efficacy while minimizing adverse effects on normal tissues. Overall, the results indicate that the SA extract, which contains ellagic acid and gallic acid as its major active constituents, exhibits potential as a time-dependent anticancer agent. It demonstrates selective cytotoxicity toward colorectal cancer cells during the early phase of treatment, consistent with previous findings reported [42,43]. However, these results were obtained from cell line experiments and may not fully reflect the complex biological responses in living organisms. For that reason, in this study, the extract was further evaluated in an animal model via oral administration. This route of administration enables the extract to undergo phase I and phase II metabolic transformations, which may lead to differences in both efficacy and safety outcomes compared with those observed in cell line experiments [44,45,46].

The in vivo xenograft model further substantiated its therapeutic potential. Xenograft models provide a physiologically relevant in vivo platform that preserves tumor heterogeneity and spatial architecture, enabling more accurate evaluation of therapeutic responses. Tumor volume accuracy was validated using multiple methods, including calipers, micro-ultrasound, and bioluminescence imaging. Previous studies have shown that ultrasound offers more reliable and precise volume estimation than calipers alone [19,47]. Additionally, ultrasound is significantly more precise and reproducible compared to calipers for xenograft tumor volume assessment in preclinical models [19]. Bioluminescence imaging enables quantitative real-time monitoring of tumor growth and treatment response, allowing early detection, reducing animal use, and minimizing experimental variability in studies [48]. In tumor xenograft models, a bioluminescence signal at least 3- to 5-fold higher than background is generally regarded as a true positive [25]. Treatment with SA consistently demonstrated superior efficacy compared to the standard chemotherapeutic agent 5-fluorouracil (5FU) in inhibiting tumor growth. Previously reported studies of ellagic acid and gallic acid have been shown to suppress tumor growth in colorectal cancer xenograft models [49,50].

Interestingly, the SA500 dose produced more pronounced tumor suppression than the higher dose (SA1000), as evidenced by greater reductions in tumor volume and cell viability from day 14 to day 21 of treatment. Under physiological conditions, chemotherapeutic efficacy in colorectal cancer is strongly influenced by drug uptake mechanisms, which involve both passive diffusion and carrier-mediated transport via monocarboxylate transporters (MCTs), organic anion transporters (OATs), and organic anion-transporting polypeptides (OATPs) [51,52,53]. Phenolic compounds such as ellagic acid and gallic acid are likewise taken up through these pathways [54]. At higher drug concentrations, however, excessive metabolic and oxidative stress can activate stress-responsive signaling pathways, including p53, AMPK, and MAPK, leading to adaptive cellular responses that limit intracellular drug accumulation [55,56]. These responses have been associated with downregulation and internalization of influx transporters, including MCT1, OAT2, and OATP1B3, thereby reducing drug uptake and promoting chemoresistance [53,57]. In parallel, colorectal cancer cells may further restrict intracellular drug exposure through activation of ATP-dependent efflux transporters, such as P-glycoprotein (ABCB1) and MRP5 (ABCC5), which are known to be upregulated under chemotherapy-induced stress and at high drug concentrations [58,59,60,61]. Consequently, the superior antitumor efficacy observed at the moderate SA500 dose likely reflects a more favorable balance between drug uptake and efflux processes, allowing sustained intracellular availability of bioactive compounds. In contrast, higher doses may trigger stress-mediated adaptive mechanisms that attenuate cellular drug accumulation, thereby limiting therapeutic efficacy despite increased exposure.

Importantly, the integration of multiple imaging modalities strengthened the reliability of the findings. Consistent results across caliper-based tumor measurements, ultrasound-derived volumetric analyses, and bioluminescence imaging of metabolic activity collectively demonstrated the superior therapeutic efficacy of the SA-treated groups compared with the NC and 5FU groups, with the most pronounced effect observed in the SA500 group. A synergistic effect of the SA extract, containing ellagic acid and gallic acid, has been demonstrated in breast cancer cells, where their combined treatment produced significantly greater cytotoxic and pro-apoptotic effects than any individual compound alone [62]. Similarly, a walnut phenolic extract rich in ellagic acid and gallic acid has been shown to inhibit the proliferation of colon cancer cells, suggesting that the cooperative action of these phenolic constituents contributes to its overall anticancer potential [63].

In safety assessment studies, histopathological examination combined with assessments of body weight, organ weight, and hematological and blood biochemical parameters is routinely used to evaluate potential systemic toxicity [64,65]. In the present study, no abnormalities in body weight or organ weight were observed relative to the control group, indicating that administration of 5-fluorouracil (5FU, 20 mg/kg) or SA extract (500 and 1000 mg/kg) did not induce overt toxicity. Hematological and blood chemistry analyses further supported the favorable safety profile of the SA extract. Consistent with previous reports, 5FU treatment was associated with mild reductions in red blood cell, hemoglobin, hematocrit, and platelet counts [66,67,68]. In contrast, animals treated with SA500 maintained hematological parameters comparable to those of the normal control group, while the SA1000 group showed only a slight reduction in platelet count. The markers of hepatic and renal function remained within normal ranges across all groups, with SA500 exhibiting the most favorable biochemical profile, including lower AST, ALT, BUN, and creatinine levels. Histopathological examination of liver tissue revealed preserved hepatic architecture in all treatment groups, further confirming the absence of hepatotoxicity. Collectively, these findings demonstrate that the SA extract is remarkably safe, even at doses 25–50 times higher than those of 5-fluorouracil (5FU), highlighting its favorable toxicological profile for potential therapeutic application.

Ex vivo analyses have demonstrated that approximately 0.3% to 9% of patients with colorectal cancer develop brain metastases [69]. Interestingly, brain metastasis was detected only in the 5FU–treated group, whereas no such occurrence was observed in either the SA-treated or NC groups as shown in Supplementary Figures S6 and S7. This observation aligns with previous evidence indicating that 5-fluorouracil (5FU) and its metabolites can elicit oxidative stress, endothelial damage, and neuroinflammatory responses, consequently compromising the structural and functional integrity of the blood–brain barrier (BBB) and increasing vascular permeability, thereby creating a permissive microenvironment for tumor cell extravasation into the brain [70]. Interestingly, these findings suggest that the SA extract, particularly at the 500 mg/kg dose, not only suppresses primary tumor growth but also limits secondary metastatic dissemination, highlighting its potential as a multifaceted therapeutic candidate for colorectal cancer. Similarly, a recent study by Chi-Chou Huang et al. (2024) reported that gallic acid significantly inhibited the migration and invasion of colorectal cancer cells, suggesting its potential role in suppressing metastatic progression [32].

Immunohistochemical (IHC) and TUNEL analyses provide valuable in situ evidence of protein expression changes, supporting mechanistic interpretations related to proliferation, apoptosis, or angiogenesis. IHC is a core technique in tumor characterization and biomarker analysis for clinical diagnostics, especially in colorectal cancer. It is widely used not only in experimental research but is also a standard approach in pathology for routine evaluation of patient biopsy specimens [71,72,73]. These methods are vital for assessing tumor proliferation and angiogenesis, linking preclinical data to clinical relevance. The TUNEL assay detects apoptotic DNA fragmentation in tissue, providing quantitative spatial data on apoptosis in both research and pathology [74,75]. In our study, tumor cell proliferation was significantly suppressed, as evidenced by reduced Ki-67 expression, with the most pronounced decrease observed in the SA1000 group, reaching levels comparable to the NC and 5FU groups. Likewise, Bcl-2 expression, an anti-apoptotic marker, was markedly downregulated in both the SA1000 and 5FU groups, whereas a moderate reduction was observed in the SA500 group. Angiogenesis, evaluated by CD31 staining, was attenuated in both SA-treated and 5FU groups, and apoptotic activity, assessed by TUNEL assay, was comparably elevated across both SA doses. At higher concentrations, such as SA1000, the extract appeared to exert more direct effects on tumor cell–intrinsic pathways, particularly those governing proliferation and apoptosis, as evidenced by the lower expression of Ki-67 and Bcl-2. However, no significant differences were observed in CD31 or TUNEL staining between the 500 and 1000 mg/kg doses. This finding suggests that increasing the dose from 500 to 1000 mg/kg may not further enhance the overall antitumor response, likely due to dose-dependent modulation of cellular transport mechanisms. Taken together with the present immunohistochemical findings, these results suggest that the phenolic-rich SA extract exerts its in vivo antitumor activity through the coordinated regulation of cell proliferation, apoptosis, and drug transport processes. Such multimodal actions may underline the greater therapeutic efficacy observed at the moderate dose (SA500), where the balance between cellular uptake and efflux remains optimal. Consistent with this finding, several phenolic constituents identified in the SA extract have been reported to exhibit antiproliferative and pro-apoptotic activities in colorectal cancer models. Gallic acid similarly reduces tumor volume and Ki-67 expression in vivo [76]. Likewise, ellagic acid has been demonstrated to downregulate Bcl-2 expression and promote apoptosis in colorectal cancer cells [31]. These observations further support that the antitumor efficacy of the SA extract is largely mediated by its phenolic components, which collectively modulate key signaling pathways involved in proliferation and apoptosis.

Furthermore, metabolomics provides a comprehensive biochemical snapshot by analyzing small-molecule metabolites, revealing cellular processes and system-wide physiological changes related to disease, treatment, or environmental effects. With lipid metabolism, membranes, and their components, acetyl-L-carnitine levels increase in tumors, marking mitochondrial stress and enhanced fatty acid transport, while their reduction after treatment indicates improved mitochondrial function and decreased metabolic stress [77]. Related to the increase of palmitoylcarnitine after treatment with 5FU or SA extracts, it could induce apoptosis in cancer cells [78,79]. Stearic acid, known to suppress tumor growth and induce apoptosis via endoplasmic reticulum stress and DNA damage, shows restoration after either 5FU or SA extract treatments, suggesting reduced tumor progression and normalized lipid metabolism [80]. 6-hydroxycaproic acid declines with tumor progression, reflecting increased fatty acid oxidation and mitochondrial dysfunction. Its anti-inflammatory effects, such as lowering IL-6 and TNF-α, may help suppress colorectal cancer and metastasis [81]. Cis-7-hexadecenoic acid supports lipid remodeling, restoring homeostasis and promoting anti-inflammatory effects [82] that may suppress tumor growth and improve outcomes [83,84]. These findings highlight the therapeutic potential of modulating fatty acid metabolism in colorectal cancer management.

Moreover, palmitoyl sphingomyelin levels are reduced in colorectal cancer due to altered sphingolipid metabolism, compromising membrane integrity and signaling. Restoration after treatment may enhance membrane stability and promote ceramide-mediated apoptosis, improving therapeutic response and inhibiting tumor progression. Similarly, choline’s role in phospholipid synthesis underlines its importance in membrane remodeling and cancer progression. These findings are supported by evidence that targeting sphingolipid pathways can impact proliferation, apoptosis, and treatment outcomes in colon cancer [85,86]. Collectively, alterations in fatty acid metabolites present not only biomarkers of colorectal cancer progression but also potential therapeutic targets for modulating tumor metabolism.

In untreated patients, circulating leucine levels may be reduced due to increased amino acid demand by tumor tissues to support cancer growth and metabolic maintenance [87]. Conversely, effective tumor suppression is generally associated with increased circulating leucine levels, as reduced tumor burden decreases amino acid utilization and tumor uptake [88,89]. Consistent with our finding, serum leucine levels were elevated following 5FU treatment. In contrast, SA treatment exhibited a distinct metabolic profile, characterized by reduced serum leucine levels despite comparable tumor suppression. This discrepancy may be attributed to the nature of SA as a crude extract containing multiple bioactive compounds, rather than a single chemotherapeutic agent such as 5FU. Previous studies have shown that phenolic compounds, including eugenol, quercetin, and gallic acid, have been reported to modulate leucine levels or leucine-related amino acid metabolic pathways [90,91,92]. The observed reduction in serum leucine following SA treatment may therefore reflect the addition or synergistic effects of multiple compounds rather than the action of a single dominant compound. Moreover, post-treatment decreases in creatine in all conditions reflect altered energy metabolism and tumor suppression. This duality highlights creatine’s complex role in cancer progression and response to therapy. In colorectal cancer, decreased malic acid results from metabolic reprogramming; 5FU and SA restore its levels, supporting malate’s function as a PKM2 regulator and potential tumor suppressor [93]. Therefore, amino acid metabolism profoundly influences colorectal cancer progression and immune modulation, with altered amino acid levels supporting tumor growth, immune evasion, and therapy resistance, highlighting potential targets for precision treatment. In our study, colorectal cancer exhibited low uracil levels. However, treatment with 5FU and SA extracts significantly increased uracil concentrations, indicating a beneficial modulation of pyrimidine metabolism. This aligns with prior research showing that uracil enhancement improves therapeutic efficacy by inhibiting dihydropyrimidine dehydrogenase and increasing 5FU bioavailability, which enhances treatment outcomes while potentially reducing toxicity [94,95,96]. These findings emphasize the value of uracil as a metabolic biomarker and therapeutic modulator in colorectal cancer management. Metabolomic analysis of this study highlights critical metabolic alterations and therapeutic impacts, underscoring metabolomics’ value in understanding colorectal cancer progression and treatment response.

By integrating the findings from both in vitro and in vivo experiments, the present study supports the potential of the phenolic-rich SA extract as a promising anticancer agent for colorectal cancer. Notably, under the current experimental conditions, the extract demonstrated tumor-suppressive effects comparable to or exceeding those of 5-fluorouracil (5-FU). Nevertheless, the dose-dependent differences in efficacy underscore the need for further investigation to optimize dosing regimens, elucidate its pharmacokinetic behavior, and define the molecular mechanisms underlying its antitumor effects. Further validation in orthotopic or intestinal xenograft models, together with the development of optimized or adjunctive formulations in combination with other natural compounds or conventional chemotherapeutic agents, may further enhance translational relevance while minimizing off-target cytotoxicity in normal tissues.

## 5. Conclusions

Comprehensive phytochemical characterization of the hydrodistillation residue extract of *Syzygium aromaticum* (SA) confirmed the presence of major phenolic constituents, gallic acid and ellagic acid, known for their antioxidant and anticancer properties. Based on this phytochemical foundation, the present study demonstrated that SA exhibits potent anticancer efficacy against colorectal cancer both in vitro and in vivo. In cell-based assays, SA inhibited colorectal cancer cell proliferation in a dose- and time-dependent manner, exhibiting selective cytotoxicity toward tumor cells at early exposure periods. Consistent with the in vitro findings, in vivo administration of SA markedly suppressed tumor growth. The 500 mg/kg dose (SA500) showed greater overall therapeutic efficacy than 5-fluorouracil (5FU) by reducing tumor volume, metabolic activity, and metastatic incidence while maintaining an excellent safety profile. At the molecular level, the higher dose (SA1000) induced stronger inhibition of cell proliferation and anti-apoptotic signaling, as reflected by lower Ki-67 and Bcl-2 expression, whereas angiogenesis (CD31) and apoptosis (TUNEL) were comparably affected in both SA-treated groups. Together, these outcomes suggest that while SA1000 exerts more direct effects on tumor cell–intrinsic pathways, SA500 achieves superior systemic efficacy, likely due to an optimal balance between cellular uptake and efflux that maintains effective intratumoral concentrations of bioactive phenolics. Furthermore, supporting these mechanistic observations, metabolomic profiling revealed distinct metabolic alterations between treated and control groups. Both SA500 and SA1000 exhibited metabolomic profiling and revealed alterations in four major metabolic pathways, fatty acid/lipid, amino acid and energy metabolism, and pyrimidine metabolism, signatures closely resembling those of 5FU, indicating comparable metabolic reprogramming associated with tumor suppression. These metabolic shifts further emphasize the systemic influence of SA on tumor metabolism in vivo.

Collectively, these findings provide strong evidence that SA extract is a promising natural product, a derived therapeutic candidate for colorectal cancer, which combines potent efficacy with improved tolerability compared with conventional chemotherapy. Further investigations into the pharmacokinetic behavior, safety profile, and underlying molecular mechanisms of the SA extract, particularly through validation in orthotopic or intestinal xenograft models, will be essential to optimize dosing strategies and to further explore its translational potential, either as a standalone agent or as an adjunctive therapy. In parallel, bioassay-guided fractionation of the SA extract should be undertaken to identify the bioactive constituents for future product development.

## Figures and Tables

**Table 1:** Major components in SA extract identified by LC-MS/MS.

Peak No.	Retention Time (min)	Mode	Parent Ion (MS ^1^)	Fragment Ion (MS ^2^)	Results
1	2.61	Negative	169.0141	125.0245	Gallic acid C_7_H_6_O_5_, MW = 170 Calc for [M−H]^−^ = 169.01315 Calc for [M+H]^+^ = 171.02880
		Positive	171.0285	153.0183, 127.0389, 125.0232, 109.0283, 107.0126, 81.0334, 53.0385, 51.0229	
2	7.41	Negative	353.0869	233.0457, 205.0508	Biflorin or isobiflorin C_16_H_18_O_9_, MW = 354 Calc for [M−H]^−^ = 353.08671 Calc for [M+H]^+^ = 355.10236
		Positive	355.1018	337.0911, 319.0811, 301.0712, 289.0711, 273.0754, 259.0601, 245.0807, 235.0601, 205.0496, 177.0544	
3	7.73	Negative	353.0870	263.0564, 245.0456, 233.0457, 205.0507	Biflorin or isobiflorin C_16_H_18_O_9_, MW = 354 Calc for [M−H]^−^ = 353.08671 Calc for [M+H]^+^ = 355.10236
		Positive	355.1019	337.0924, 319.0809, 301.0709, 283.0604, 271.0603, 259.0601, 245.0805, 235.0599, 205.0495, 189.0553	
4	8.72	Negative	275.0196	257.0094, 229.0144, 219.0309, 203.0351	Unknown C_13_H_8_O_7_, MW = 276 Calc for [M−H]^−^ = 275.01863 Calc for [M+H]^+^ = 277.03428 Calc for [M+Na]^+^ = 299.01622
		Positive	277.0341	259.0235, 231.0288, 215.0338, 203.0344, 187.0389	
			299.0162	283.0490, 281.0514, 268.9973, 224.9489, 153.0184, 135.2761, 110.3592	
5	10.07	Negative	300.9986	283.9956, 257.0068, 245.0098, 229.0145, 201.0194, 185.0239	Ellagic acid C_14_H_6_O_8_, MW = 302 Calc for [M−H]^−^ = 300.99789 Calc for [M+H]^+^ = 303.01354 Calc for [M+Na]^+^ = 344.99549
		Positive	303.0132	285.0028, 275.0188, 257.0079, 247.0234, 83.0384, 63.0229	
			324.9954	n/a	

**Table 2:** IC_50_ values (µg/mL) of the SA extract in HCT116-Red-FLuc and MRC-5 cells were determined following 24, 48, and 72 h of treatment. The selectivity index (SI) was calculated as the ratio of the IC_50_ value in the normal cell line to that in the colorectal cancer cell line at each corresponding time point.

Time (h)	HCT116-Red-FLuc IC_50_ (µg/mL)	MRC-5 Cells IC_50_ (µg/mL)	SI
24 h	47.16 ± 4.39	204.78 ± 47.39	4.41
48 h	46.61 ± 6.01	80.09 ± 11.82	1.72
72 h	44.12 ± 2.99	58.33 ± 4.95	1.32

Data are expressed as mean ± SD (*n* = 3). The IC_50_ values were derived from dose–response curves generated by nonlinear regression analysis.

**Table 3:** Hematology and Blood Chemical Parameters of Mice on Day 21 after Treatment with NC, 5FU, SA500 and SA1000.

Blood Parameters	Groups				Normal Range [29]
	NC	5FU	SA500	SA1000	
WBC (×10^3^/μL)	2.53 ± 0.71	2.66 ± 2.14	3.50 ± 1.24	2.90 ± 0.95	3.10–13.04
RBC (×10^6^/μL)	9.68 ± 1.48	8.18 ± 0.56	9.20 ± 1.28	8.86 ± 1.38	8.83–11.73
HGB (g/dL)	15.60 ± 2.27	13.12 ± 0.60	14.50 ± 1.65	14.10 ± 1.92	12.7–18.4
HCT (%)	46.20 ± 5.02	42.40 ± 1.67	44.20 ± 5.50	43.60 ± 3.36	49.6–66.0
PLT (×10^3^/μL)	834.80 ± 21.57	756.60 ± 195.11 *	825.00 ± 32.86	613.00 ± 413.85 **	558–1564
Plasma protein (g/dL)	6.00 ± 0.00	6.00 ± 0.00	6.00 ± 0.00	6.00 ± 0. 35	-
Creatinine (mg/dL)	0.43 ± 0.18	0.46 ± 0.16	0.40 ± 0.07	0.37 ± 0.09	0.3–0.5
BUN (mg/dL)	27.12 ± 4.87	28.90 ± 3.41	24.56 ± 2.33	26.60 ± 4.54	18–45
AST (U/L)	170.80 ± 56.41	106.20 ± 36.80 ***	112.00 ± 59.03 ***	157.40 ± 71.47	50–215
ALT (U/L)	40.00 ± 4.53	37.20 ± 5.07	33.00 ± 7.07	41.40 ± 10.09	27–78
ALP (U/L)	59.20 ± 14.31	40.80 ± 14.96	45.80 ± 11.76	40.40 ± 16.38	63–178

WBC: White Blood Cell count, RBC: Red Blood Cell count, HGB: Hemoglobin, HCT: Hematocrit, PLT: Platelet count, BUN: blood urea nitrogen, AST: aspartate aminotransferase, ALT: alanine aminotransferase, ALP: Alkaline Phosphatase. Values are the mean ± SD (*n* = 5). Statistical significance versus NC: * *p* < 0.05, ** *p* < 0.01, *** *p* < 0.001.

## Data Availability

The data from this study can be provided on request by the corresponding author.

## Supplementary Materials

The following supporting information can be downloaded at: https://www.mdpi.com/article/10.3390/pharmaceutics18010079/s1, Figure S1. Mass spectra of gallic acid (1) at retention time of 2.61 min (A) full scan at 100–1000 *m*/*z* in negative mode, (B) fragment mass of parent ion at *m*/*z* 169.0141 in negative mode, (C) full scan at 100–1000 *m*/*z* in positive mode, and (D) fragment mass of parent ion at *m*/*z* 171.0266 in positive mode. Figure S2. Mass spectra of bioflorin or isobioflorin (2) at retention time of 7.41 min (A) full scan at 100–1000 *m*/*z* in negative mode, (B) fragment mass of parent ion at *m*/*z* 353.0869 in negative mode, (C) full scan at 100–1000 *m*/*z* in positive mode, and (D) fragment mass of parent ion at *m*/*z* 355.1018 in positive mode. Figure S3. Mass spectra of bioflorin or isobioflorin (3) at retention time of 7.73 min (A) full scan at 100–1000 *m*/*z* in negative mode, (B) fragment mass of parent ion at *m*/*z* 353.0870 in negative mode, (C) full scan at 100–1000 *m*/*z* in positive mode, and (D) fragment mass of parent ion at *m*/*z* 355.1019 in positive mode. Figure S4. Mass spectra of unidentified compound (4) at retention time of 8.72 min (A) full scan at 100–1000 *m*/*z* in negative mode, (B) fragment mass of parent ion at *m*/*z* 275.0197 in negative mode, (C) full scan at 100–1000 *m*/*z* in positive mode, (D) fragment mass of parent ion at *m*/*z* 277.0341 in positive mode, and (E) fragment mass of parent ion at *m*/*z* 299.0161 in positive mode. Figure S5. Mass spectra of ellagic acid (5) at retention time of 10.08 min (A) full scan at 100–1000 *m*/*z* in negative mode, (B) fragment mass of parent ion at *m*/*z* 300.9986 in negative mode, (C) full scan at 100–1000 *m*/*z* in positive mode, and (D) fragment mass of parent ion at *m*/*z* 303.0132 in positive mode. Figure S6. Mapping organs of ex vivo showed metastasis in vital organs. Figure S7. Ex vivo showed inhibit metastasis by of SA extract.

## Abbreviations

The following abbreviations are used in this manuscript:

SA	*Syzygium aromaticum* hydrodistillation residue extract
IC_50_	the half-maximal inhibitory concentration
FOLFOX	Folinic acid (leucovorin), Fluorouracil (5FU) and Oxaliplatin
FOLFIRI	Folinic acid (leucovorin), Fluorouracil (5FU) and Irinotecan
