# Supplementary material for: Antitumor Activity of the Ethanolic Extract from Syzygium aromaticum in Colorectal Cancer Xenograft Mice
Source: Pharmaceutics. 2026 Jan 7;18(1):79. doi: 10.3390/pharmaceutics18010079 (PMC12844892; doi:10.3390/pharmaceutics18010079)
Supplement: Supplementary file 1 [file pharmaceutics-18-00079-s001.zip › pharmaceutics-4024617-supplementary.pdf]

pharmaceutics

MDPI

Supplementary material

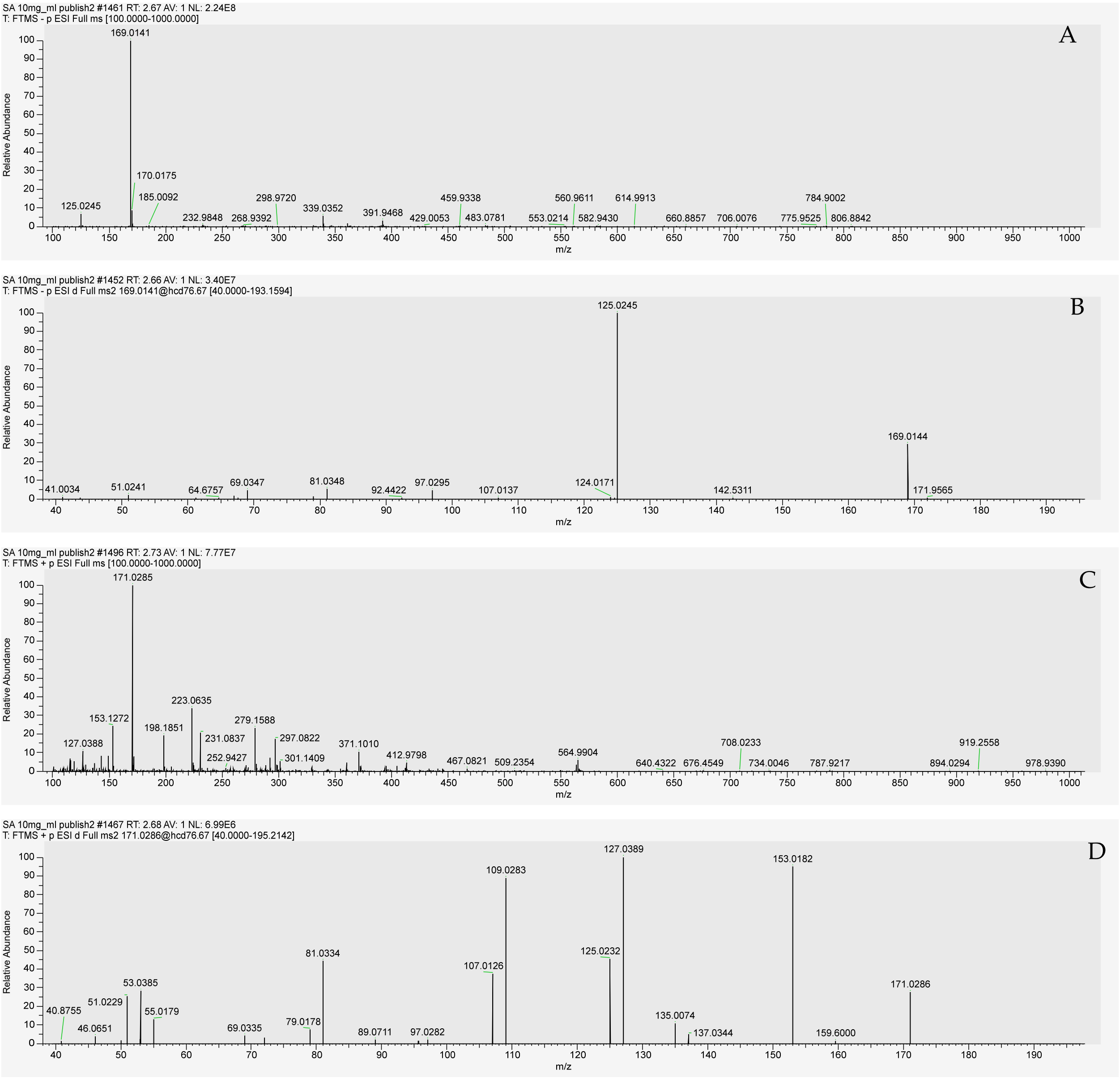

Figure S1. Mass spectra of gallic acid (**1**) at retention time of 2.61 min (A) full scan at 100-1000 *m*/*z* in negative mode, (B) fragment mass of parent ion at *m*/*z* 169.0141 in negative mode, (C) full scan at 100-1000 *m*/*z* in positive mode, and (D) fragment mass of parent ion at *m*/*z* 171.0266 in positive mode.

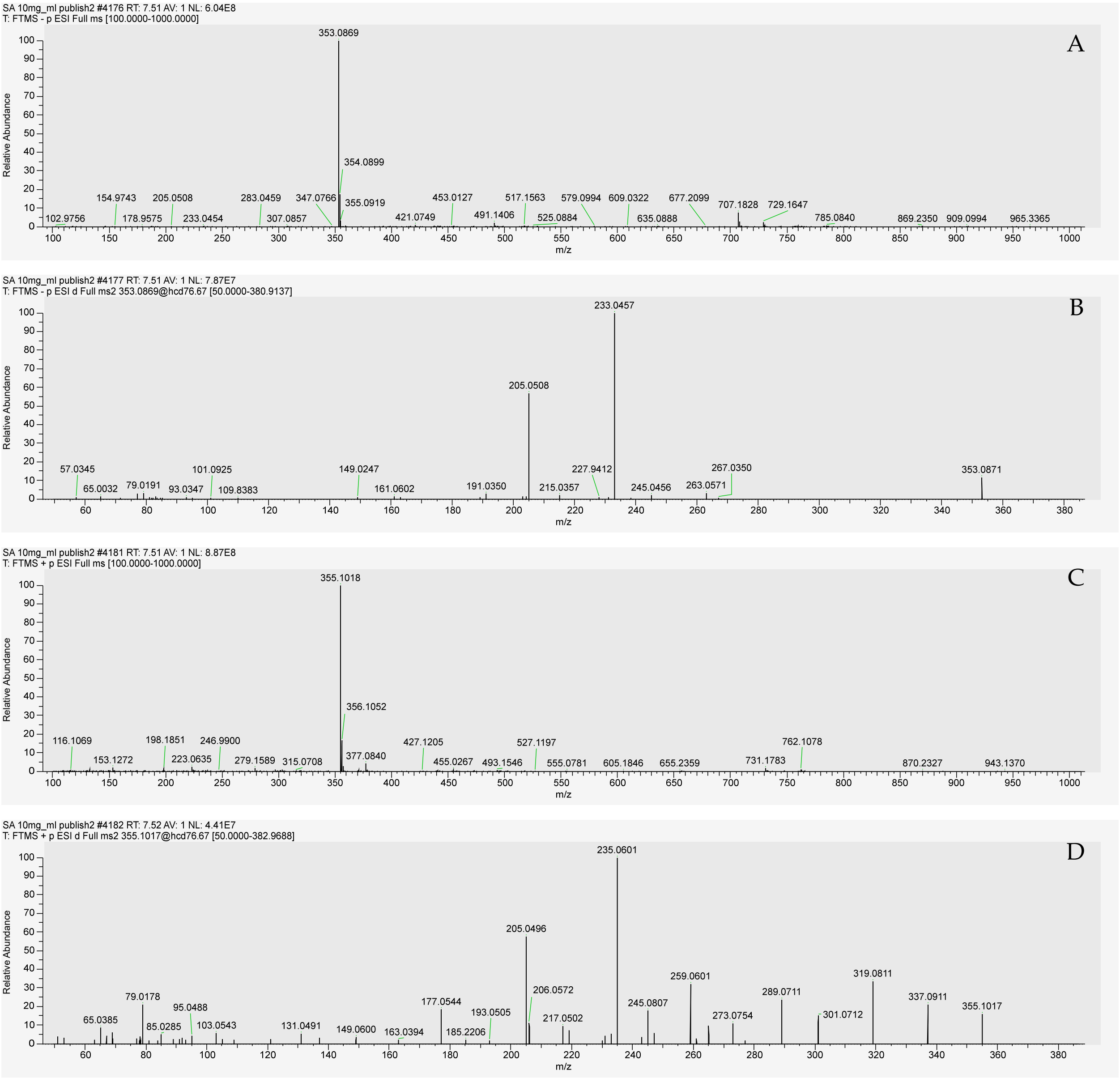

Figure S2. Mass spectra of bioflorin or isobioflorin (**2**) at retention time of 7.41 min (A) full scan at 100-1000 *m/z* in negative mode, (B) fragment mass of parent ion at *m/z* 353.0869 in negative mode, (C) full scan at 100-1000 *m/z* in positive mode, and (D) fragment mass of parent ion at *m/z* 355.1018 in positive mode.

https://doi.org/10.3390/pharmaceutics18010079

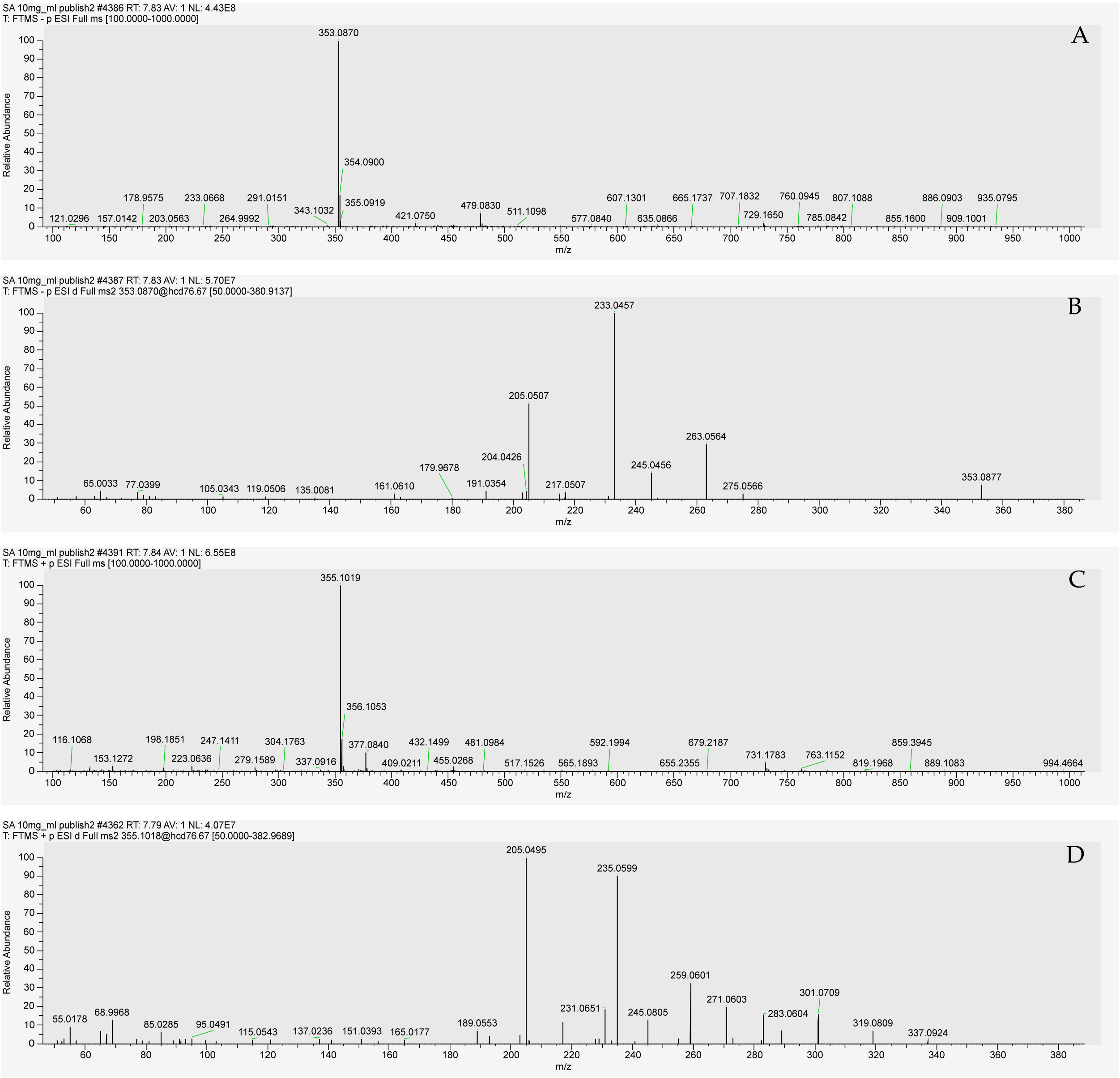

Figure S3. Mass spectra of bioflorin or isobioflorin (**3**) at retention time of 7.73 min (A) full scan at 100-1000 *m/z* in negative mode, (B) fragment mass of parent ion at *m/z* 353.0870 in negative mode, (C) full scan at 100-1000 *m/z* in positive mode, and (D) fragment mass of parent ion at *m/z* 355.1019 in positive mode.

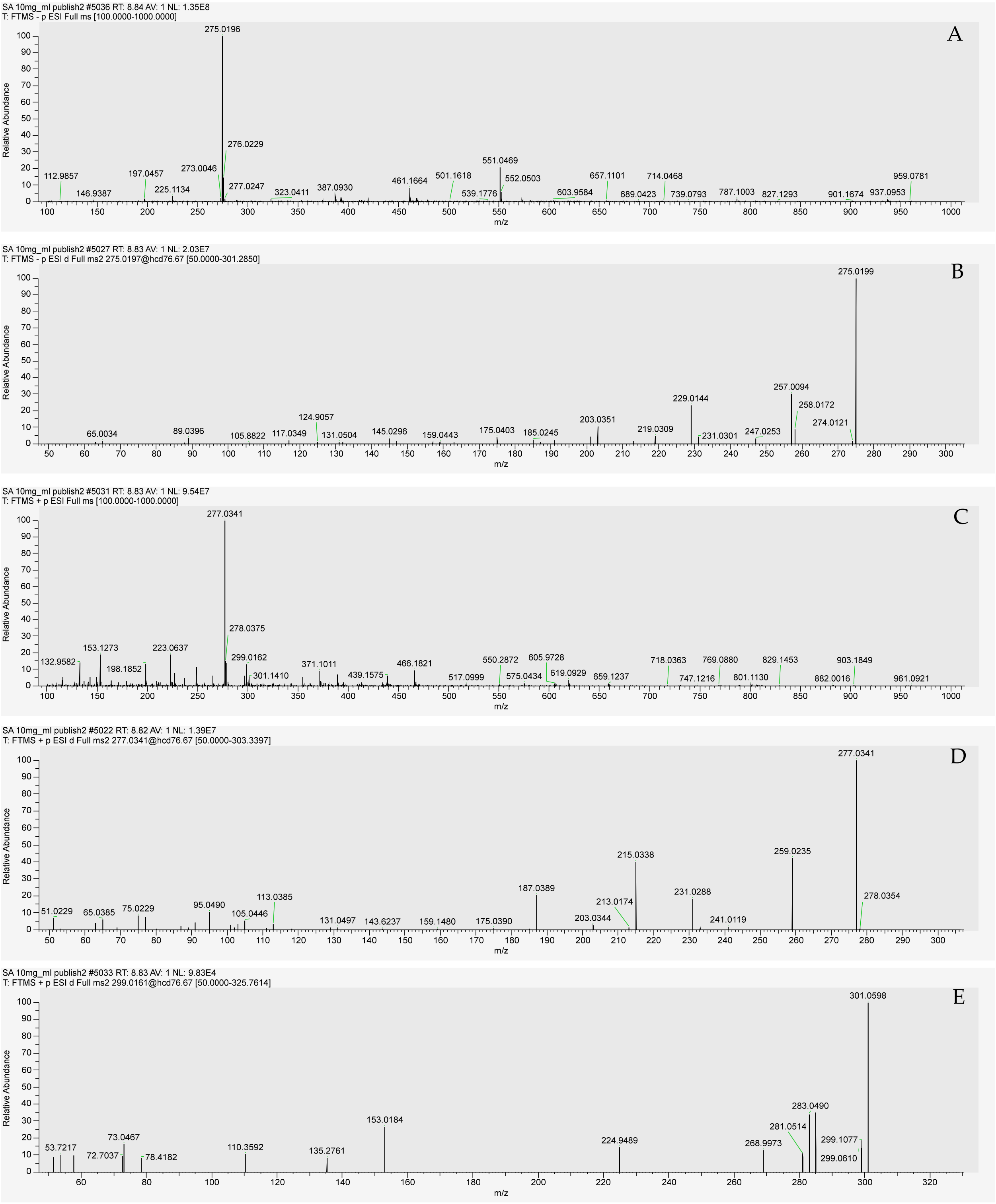

Figure S4. Mass spectra of unidentified compound (**4**) at retention time of 8.72 min (A) full scan at 100-1000 *m/z* in negative mode, (B) fragment mass of parent ion at *m/z* 275.0197 in negative mode, (C) full scan at 100-1000 *m/z* in positive mode, (D) fragment mass of parent ion at *m/z* 277.0341 in positive mode, and (E) fragment mass of parent ion at *m/z* 299.0161 in positive mode.

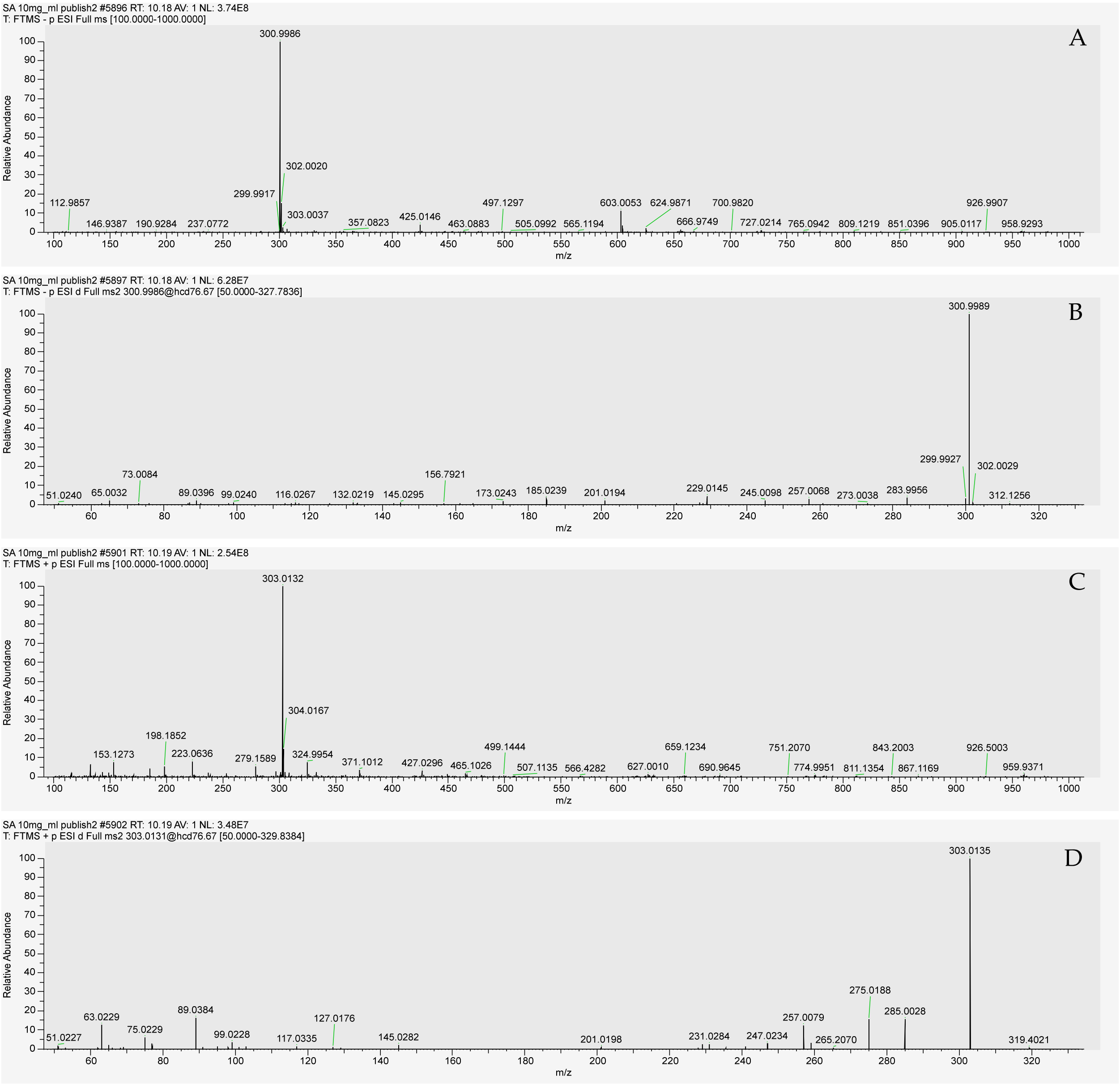

Figure S5. Mass spectra of ellagic acid (**5**) at retention time of 10.08 min (A) full scan at 100-1000 *m/z* in negative mode, (B) fragment mass of parent ion at *m/z* 300.9986 in negative mode, (C) full scan at 100-1000 *m/z* in positive mode, and (D) fragement mass of parent ion at *m/z* 303.0132 in positive mode.

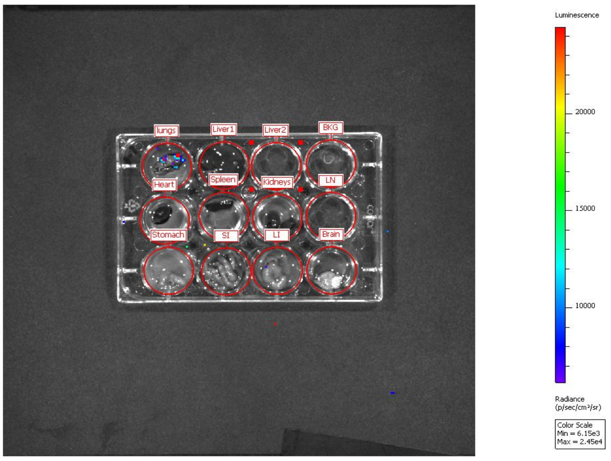

Figure S6: Mapping organs of ex vivo showed metastasis in vital organs.

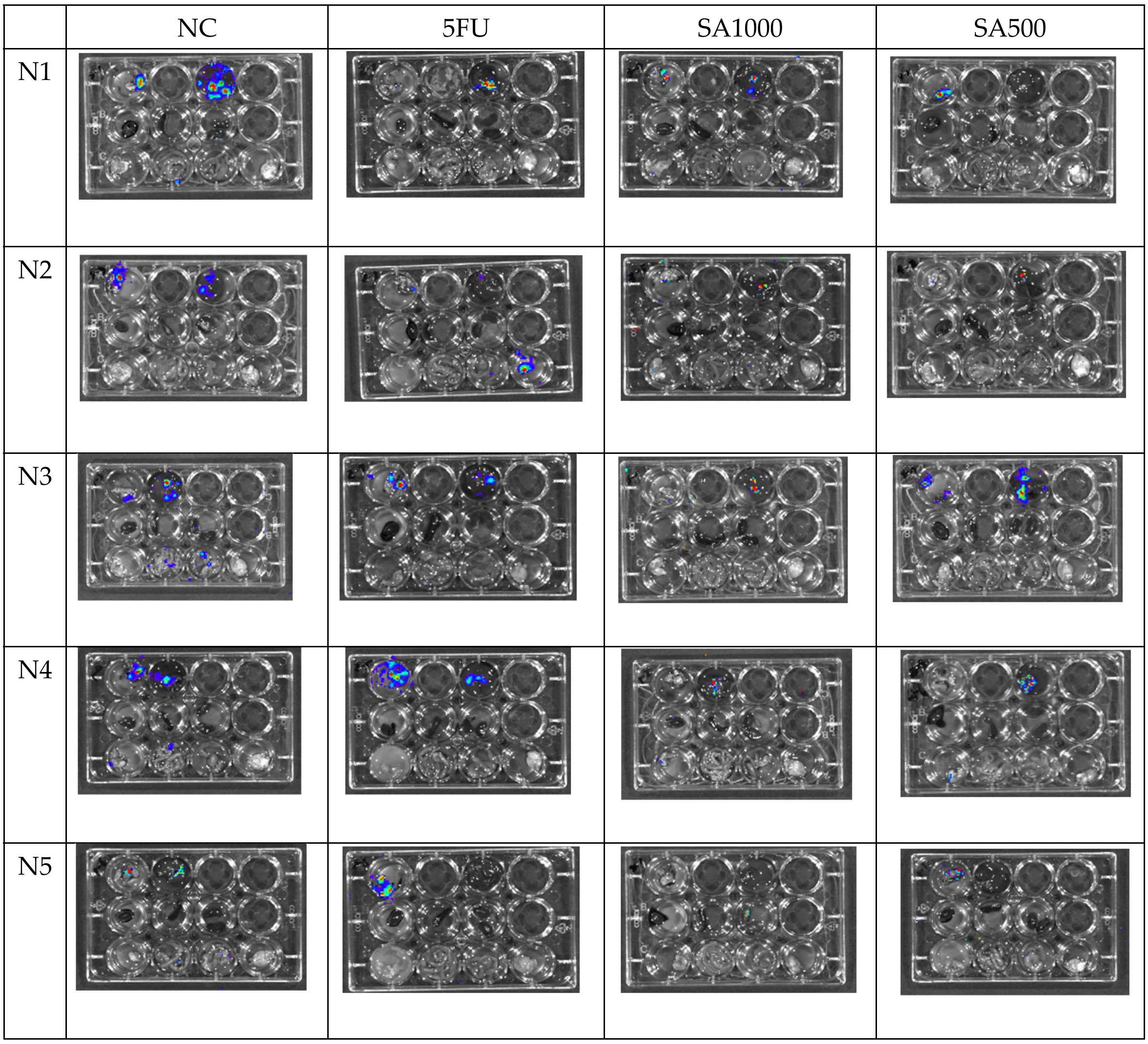

Figure S7: Ex vivo showed inhibit metastasis by of SA extract.

Disclaimer/Publisher's Note: The statements, opinions and data contained in all publications are solely those of the individual author(s) and contributor(s) and not of MDPI and/or the editor(s). MDPI and/or the editor(s) disclaim responsibility for any injury to people or property resulting from any ideas, methods, instructions or products referred to in the content.
